# Long noncoding RNA LEENE promotes angiogenesis and ischemic recovery in diabetes models

**DOI:** 10.1172/JCI161759

**Published:** 2022-12-13

**Authors:** Xiaofang Tang, Yingjun Luo, Dongqiang Yuan, Riccardo Calandrelli, Naseeb Kaur Malhi, Kiran Sriram, Yifei Miao, Chih-Hong Lou, Walter Tsark, Alonso Tapia, Aleysha T. Chen, Guangyu Zhang, Daniel Roeth, Markus Kalkum, Zhao V. Wang, Shu Chien, Rama Natarajan, John P. Cooke, Sheng Zhong, Zhen Bouman Chen

**Affiliations:** 1 Department of Diabetes Complications and Metabolism, Arthur Riggs Diabetes and Metabolism Research Institute, City of Hope, Duarte, California, USA.; 2 Department of Bioengineering, UCSD, La Jolla, California, USA.; 3 Irell and Manella Graduate School of Biological Sciences,; 4 Gene Editing and Viral Vector Core, and; 5 Transgenic Mouse Facility, Center for Comparative Medicine, City of Hope, Duarte, California, USA.; 6 Department of Diabetes and Cancer Metabolism and; 7 Department of Immunology & Theranostics, Arthur Riggs Diabetes and Metabolism Research Institute, Center for Comparative Medicine, City of Hope, Duarte, California, USA.; 8 Department of Medicine, UCSD, La Jolla, California, USA.; 9 Department of Cardiovascular Sciences, Houston Methodist Research Institute, Houston, Texas, USA.

**Keywords:** Angiogenesis, Vascular biology, Diabetes, Endothelial cells, Noncoding RNAs

## Abstract

Impaired angiogenesis in diabetes is a key process contributing to ischemic diseases such as peripheral arterial disease. Epigenetic mechanisms, including those mediated by long noncoding RNAs (lncRNAs), are crucial links connecting diabetes and the related chronic tissue ischemia. Here we identify the lncRNA that enhances endothelial nitric oxide synthase (eNOS) expression (LEENE) as a regulator of angiogenesis and ischemic response. LEENE expression was decreased in diabetic conditions in cultured endothelial cells (ECs), mouse hind limb muscles, and human arteries. Inhibition of LEENE in human microvascular ECs reduced their angiogenic capacity with a dysregulated angiogenic gene program. Diabetic mice deficient in *Leene* demonstrated impaired angiogenesis and perfusion following hind limb ischemia. Importantly, overexpression of human LEENE rescued the impaired ischemic response in *Leene*-knockout mice at tissue functional and single-cell transcriptomic levels. Mechanistically, LEENE RNA promoted transcription of proangiogenic genes in ECs, such as *KDR* (encoding VEGFR2) and *NOS3* (encoding eNOS), potentially by interacting with LEO1, a key component of the RNA polymerase II–associated factor complex and MYC, a crucial transcription factor for angiogenesis. Taken together, our findings demonstrate an essential role for LEENE in the regulation of angiogenesis and tissue perfusion. Functional enhancement of LEENE to restore angiogenesis for tissue repair and regeneration may represent a potential strategy to tackle ischemic vascular diseases.

## Introduction

Angiogenesis is a unique function of microvascular endothelial cells (MVECs) that involves highly orchestrated interactions among growth factors, membrane receptors, and signaling molecules (1, 2). While physiological stimuli, e.g., hypoxia, may promote angiogenesis, pathological conditions such as diabetes can provoke endotheliopathy, signified by impaired nitric oxide (NO) bioavailability and disrupted angiogenesis (3). Such impaired EC function is a crucial mechanism underlying a variety of diabetes-associated cardiovascular diseases, including peripheral arterial disease (PAD). Among multiple risk factors for PAD, diabetes is the most prominent, as it is associated with an increased risk of PAD and its most severe form, critical limb ischemia (CLI) (4). Despite this strong clinical implication, molecular underpinnings linking diabetes and vascular damage remain incompletely understood. Therapeutically, strategies aiming at restoring angiogenesis to improve tissue perfusion hold promise, but so far have been unsuccessful in treating PAD and CLI (5). A more complete understanding of angiogenesis is therefore required to provide meaningful insights toward novel therapeutics for treating this debilitating disease.

Long noncoding RNAs (lncRNAs), generally defined as ncRNA transcripts over 200 bp in length, are emerging epigenetic modulators of cardiovascular health and disease (6–10). To date, more than 30,000 lncRNAs have been annotated in the human genome. In ECs, a growing list of lncRNAs has been identified to regulate endothelial NO synthase–derived (eNOS-derived) NO bioavailability, EC permeability, and angiogenic function (11–14). However, the disease relevance of many of these lncRNAs and the underlying molecular mechanisms remain poorly defined. lncRNAs can regulate cellular functions through diverse modes of action, e.g., genomic imprinting, modifying chromatin states, acting as competing endogenous RNAs, and driving the organization of nuclear compartments (15–17). When localized in the nucleus, lncRNAs can exert their gene regulatory function through binding to chromatin and regulating gene transcription. We have observed profound changes in chromatin-associated lncRNAs in ECs exposed to high levels of glucose and TNF-α, which in part mimic diabetic conditions (18, 19).

In the present study, we aimed to identify lncRNAs that serve as epigenetic links between diabetes and impaired angiogenic function of ECs. From an unbiased screening, we found the top candidate to be LEENE (lncRNA enhancing eNOS expression), which we previously identified as a flow-inducible lncRNA transcribed from *LINC00520*, an enhancer locus in human ECs (18). LEENE expression was suppressed in cultured ECs treated with diabetes-associated stimuli (e.g., high glucose and TNF-α) in tissues from diabetic mice, and in intima from diabetic human arteries. In contrast, LEENE was induced by proangiogenic stimuli, e.g., hypoxia and ischemia. We went on to investigate LEENE in vitro and in vivo using cultured human ECs and KO mouse models, which supported an essential role of LEENE in angiogenesis and ischemic recovery especially in the context of diabetes. Finally, we profiled the LEENE interactomes and identified a mechanism by which LEENE engages LEO1 and MYC to promote the transcription of proangiogenic genes, exemplified by *KDR* (encoding VEGFR2) and *NOS3* (encoding eNOS). Collectively, our findings suggest a regulatory role of LEENE in improving diabetes-impaired angiogenesis and vascular dysfunction, which may be translationally exploited toward developing novel therapeutic avenues to treat ischemic diseases, including PAD.

## Results

### LEENE is suppressed in diabetic conditions and induced by proangiogenic stimuli in ECs.

**Figure 1:**
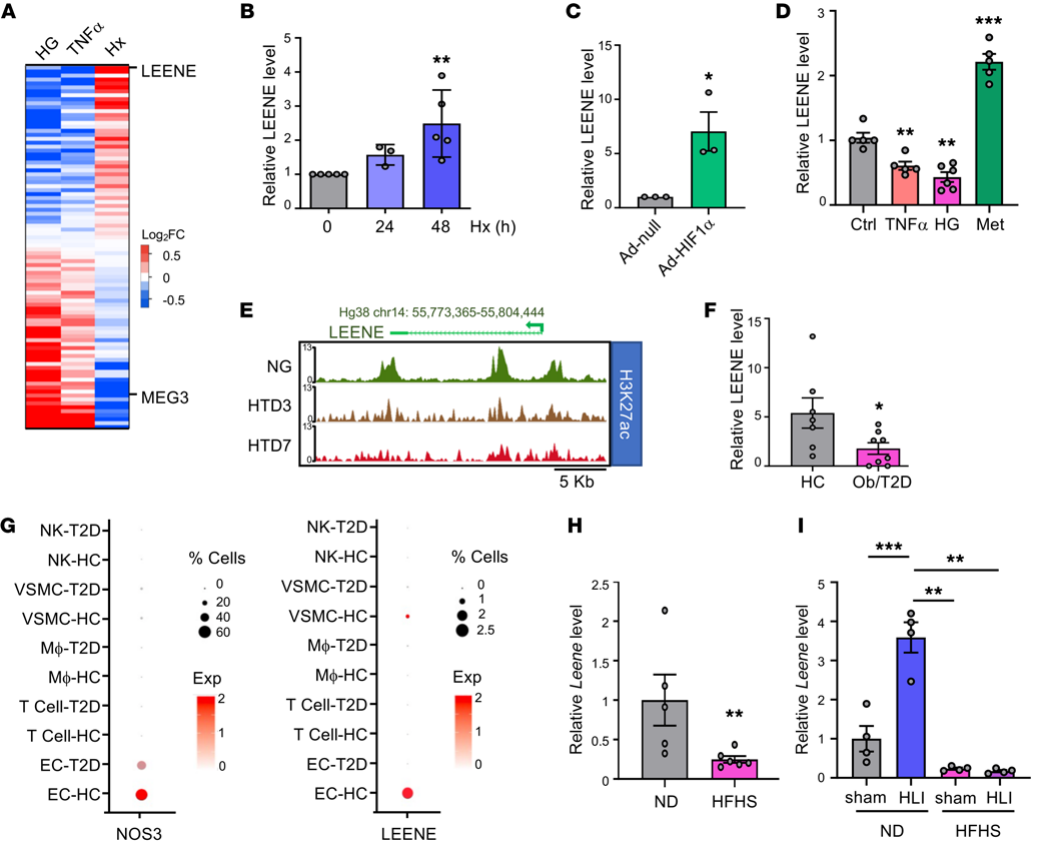
Differential regulation of LEENE by conditions that affect angiogenesis. (**A**) Heatmap of lncRNA expression in ECs, ranked by fold-change (FC) determined by RNA-seq in HUVECs treated with 25 mM glucose (high glucose [HG] vs. normal glucose [NG]/osmolarity control [ctrl]) (2 replicates), HUVECs and HAECs treated with 100 ng/mL TNF-α (vs. untreated ctrl) (1 replicate of each), and HMVECs subjected to hypoxia (Hx [2% O_2_], vs. normoxia) (2 replicates). (**B**–**D**) qPCR of *LEENE* in HMVECs subjected to normoxia (0 h) or Hx for the indicated times (**B**), infected with Ad-null or Ad-HIF1α (**C**), and treated with 100 ng/mL TNF-α, HG, or 10 mM metformin (Met) for 24 hours (**D**), with respective controls set to 1 (*n* = 3–6 biological replicates/group). (**E**) H3K27ac ChIP-seq signals in the *LEENE* locus from ECs subjected to NG or HG and 5 ng/mL TNF-α for 3 (HTD3) and 7 days (HTD7). (**F**) qPCR of *LEENE* in intima isolated from human mesenteric arteries of age-matched healthy control (HC) or donors with severe obesity (ob) and/or (pre)T2D. (**G**) Dot plot showing expression of *NOS3* and *LEENE* in HC and T2D human mesenteric arteries detected by scRNA-seq. Dot size denotes the percentage of cells expressing the corresponding gene and dot color represents the average expression. (**H** and **I**) qPCR of *Leene* in aortas from male C57BL/6J mice fed a normal chow diet (ND) or an HFHS diet starting from 8 weeks old for 16 weeks (*n* = 5–6/group) in (**H**) and in ischemic (HLI) and sham-operated control limbs (sham) of normal chow– or HFHS diet–fed male C57BL/6J mice (*n* = 7 mice/group) (**I**). Data are represented as mean ± SEM. *P < 0.05; **P < 0.01; ***P < 0.001 by 1-way ANOVA with Tukey’s post hoc test (**B** and **D**), 2-tailed Student’s *t* test (**C**, **F**, and **H**), or 2-way ANOVA with Tukey’s post hoc test (**I**).

To identify lncRNAs that may play a role in the diabetes-impaired angiogenesis in ECs, we examined RNA sequencing (RNA-seq) data sets from human ECs subjected to high glucose (HG) (19) (GSE135357), TNF-α (20) (GSE163433), and hypoxia (2% O_2_) (21) (GSE136912), respectively. We sought lncRNAs that are similarly regulated by HG and TNF-α (2 major factors associated with diabetes known to impair angiogenic function) but divergently regulated by hypoxia (a typical proangiogenic stimulus). Among the lncRNAs downregulated by HG and TNF-α but upregulated by hypoxia, LEENE ranked at the top (Figure 1A, Supplemental Figure 1, and Supplemental Table 1; supplemental material available online with this article; https://doi.org/10.1172/JCI161759DS1). In contrast, MEG3, an aging-induced and antiangiogenic lncRNA in ECs (22), exhibited the opposite pattern. As a validation, qPCR showed that LEENE was increased in human MVECs (HMVECs) by hypoxia and hypoxia-inducible factor 1α (HIF1α), a key transcription factor (TF) activated by hypoxia that induces angiogenesis (23) (Figure 1, B and C) but is decreased by TNF-α and HG (Figure 1D). Metformin, a first-line anti–type 2 diabetes (anti-T2D) drug that promotes revascularization in ischemic tissues (24), upregulated LEENE (Figure 1D). Moreover, the enrichment of H3K27ac (a marker of active enhancer and open chromatin) in the *LEENE* DNA (i.e., *LINC00520*) locus, was also decreased in ECs treated with HG and TNF-α, in line with the suppression of *LEENE* RNA levels (Figure 1E).

For human disease relevance, we examined LEENE expression in the intima freshly isolated from human mesenteric arteries. Compared with age-matched control donors (Supplemental Table 2), LEENE expression was lower in the intima from donors with severe obesity and (pre-)T2D (Figure 1F). Furthermore, in our single-cell RNA-seq (scRNA-seq) data from human mesenteric arteries (GSE135357) (19), LEENE expression, similar to that of eNOS (encoded by *NOS3*), was mainly detected in ECs and at a lower level in diabetic than control donors (Figure 1G). Of note, the T2D donors had either untreated T2D or T2D for over 10 years without compliance with treatment.

In mice, there is a Leene homolog (with an expressed sequence tag BY707159.1, later updated to Gm41148) that shares genomic features, partial sequence identity, and flow inducibility with human LEENE (18). In agreement with the human data, the levels of Leene were lower in aortae of mice with obesity and hyperglycemia induced by a high-fat, high-sucrose (HFHS) diet, as compared with animals fed a normal diet (Figure 1H). Moreover, when subjected to hind limb ischemia (HLI), the chow-fed mice expressed much higher levels of Leene in the ischemic gastrocnemius muscles. However, such induction of Leene by ischemia was abolished in mice fed an HFHS diet (Figure 1I), suggesting a potential role of LEENE in ischemic recovery that is impaired in diabetic mice (25). Collectively, these data demonstrate that LEENE is suppressed in diabetic conditions but induced by stimuli that promote angiogenesis.

### LEENE promotes angiogenesis in vitro.

**Figure 2:**
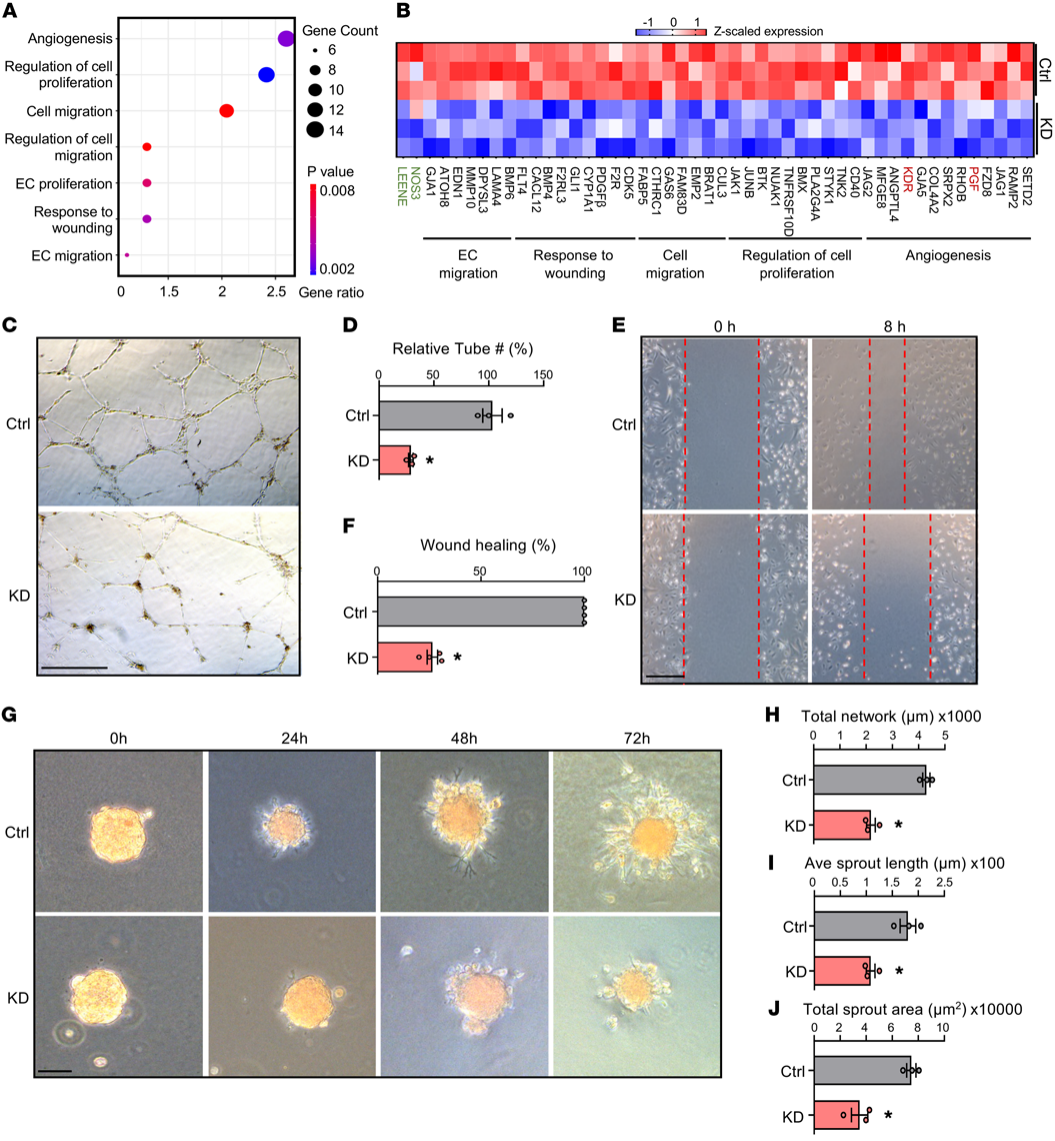
LEENE knockdown inhibits angiogenesis in vitro. (**A** and **B**) HUVECs were transfected with scramble or LEENE LNA gapmers and then subjected to pulsatile flow (12 ± 1 dyne/cm^2^) for 24 hours. Total RNA from 3 biological replicates was subjected to RNA-seq. Significantly enriched pathways related to angiogenesis in the DEGs (scramble vs. LEENE LNA) were plotted with *P* values and numbers (gene counts) and percentage of genes involved (gene ratio). (**B**) Heatmap showing expression of select DEGs involved in pathways shown in **A**. *LEENE*, *NOS3*, *KDR*, and *PGF* are colored in green and red for distinction. (**C**–**J**) HMVECs were transfected with respective LNAs for 48 hours and then used for tube formation (**C** and **D**), scratch wound (**E** and **F**), and 3D spheroid-sprouting assays (**G–J**). Scale bars: 0.5 mm (**C**), 1 mm (**E**), and 100 μm (**G**). Data are represented as mean ± SEM from 3 independent experiments. *P = 0.02 based on 2-tailed Student’s *t* test.

Next, we tested whether LEENE regulates angiogenesis. First, we inhibited LEENE and performed bulk RNA-seq to examine the LEENE-regulated transcriptional program. We knocked down (KD) LEENE using a previously validated locked nucleic acid (LNA) gapmer in HUVECs exposed to pulsatile flow, which elevates the endogenous levels of LEENE (18). RNA-seq analysis revealed that the differentially expressed genes (DEGs) were enriched for multiple angiogenesis pathways such as cell migration and proliferation and responses to wounding and VEGF (Supplemental Figure 2). Furthermore, these pathways were particularly enriched in the downregulated, rather than upregulated, DEGs (Figure 2A and Supplemental Figure 3), suggesting a positive role of LEENE in angiogenesis. Along with the decreased LEENE and eNOS RNA levels, multiple genes promoting angiogenesis and proliferation, including *KDR* (encoding VEGFR2, a major regulator promoting angiogenesis) and *PGF* (encoding placental growth factor, a potent angiogenic factor), were downregulated by LEENE KD (Figure 2B). Consistently, in HMVECs, LEENE KD reduced tube forming (Figure 2, C and D), wound closure (Figure 2, E and F), and sprouting (Figure 2, G–J) capacity. These data indicate that LEENE is a mediator in EC angiogenesis.

### Generation and baseline characterization of Leene-KO mice.

**Figure 3:**
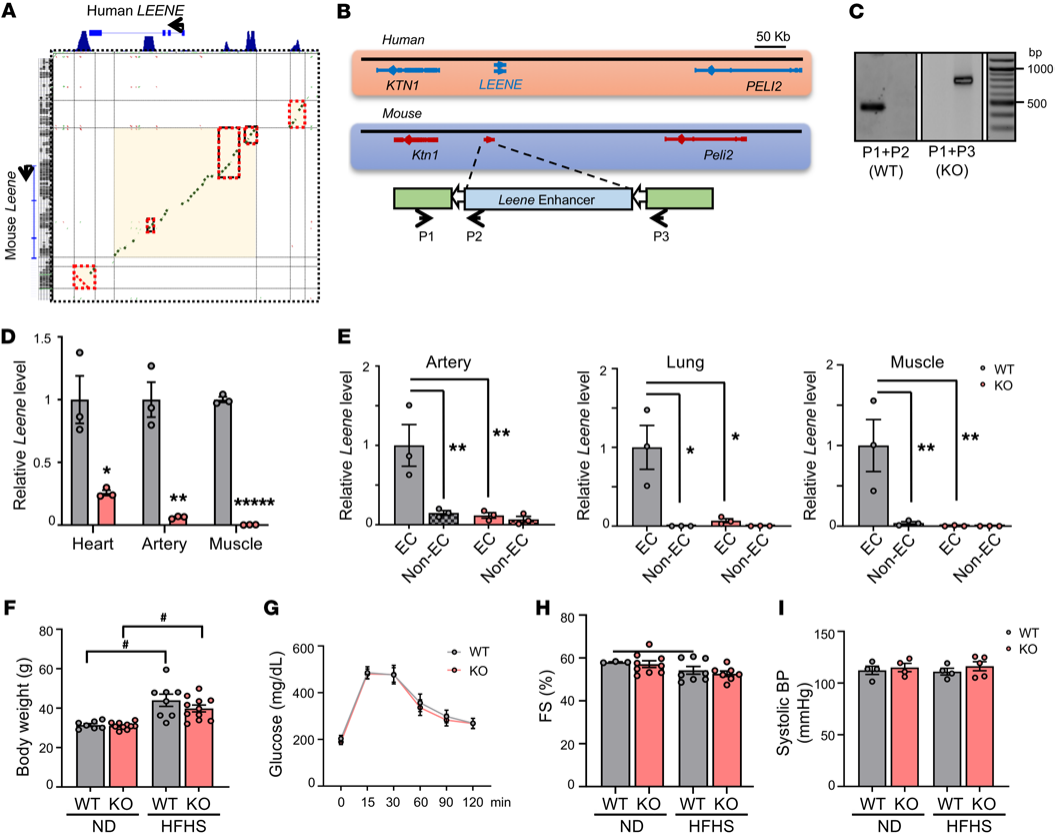
Generation of a *Leene*-KO mouse model. (**A**) DNA sequence alignment between the human *LEENE* and the syntenic region in mouse. Green indicates conservation on the sense strand and red indicates conservation on the antisense strand. Red boxes mark the sequence alignment in the H3K27ac-enriched peak regions shown on the top. (**B**) Human *LEENE* and mouse *Leene* loci; CRISPR/Cas9 targeting strategy in mouse genome and genotyping primers to identify WT (P1 + P2) and KO (P1 + P3). (**C**) Genotyping by PCR with P1–P3 primers as depicted in **B**. (**D**) qPCR of *Leene* in different organs/tissue of WT and KO littermates (*n* = 3/group). (**E**) qPCR of *Leene* in EC and non–EC-enriched fractions isolated from different tissues of WT and KO littermates (*n* = 3 mice/group). (**F**–**I**) Male *Leene*-KO and WT littermates were fed chow or HFHS diet for 16 weeks starting at 8 weeks old. (**F**) Body weight, (**G**) glucose tolerance, (**H**) fractional shortening (FS), and (**I**) systolic BP were measured (*n* = 3–11/group). Data are represented as mean ± SEM. ^#^*P* = 0.05; *P < 0.05; **P < 0.01; *****P < 0.00001 between indicated groups by 2-tailed Student’s *t* test (**D**) or 2-way ANOVA followed by Tukey’s test (**E** and **F**).

To explore the functional importance of LEENE in vivo, we generated a *Leene*-KO mouse model. Comparing the human and mouse genomic sequences, the conservation is higher in the regions marked by H3K27ac (Figure 3A). We deleted the syntenic region of human *LEENE* flanking the upstream enhancer, which we have shown to regulate the transcriptional level of LEENE (18), and coding regions in the mouse genome by using CRISPR/Cas9 editing (Figure 3B and Supplemental Figure 4). Genotyping of the resulting KO line verified the intended deletion (Figure 3C), with diminished levels of *Leene* RNA in multiple tissues, including the heart, skeletal muscle, and aorta (Figure 3D). Consistently, in EC-enriched fractions isolated from these tissues, *Leene* expression was ablated, while the non-EC fractions expressed marginal levels of *Leene* in both WT and KO mice (Figure 3E).

The *Leene-*KO mice were born at Mendelian ratios, and no overt abnormality was observed in their viability, fertility, breeding, and development (Supplemental Tables 3 and 4). In terms of metabolic parameters, KO and WT littermates had comparable body weight and composition as well as similar glucose levels under both normal chow and HFHS diet (Figure 3, F and G, and Supplemental Figures 5 and 6). Cardiac function and BP were also comparable between WT and KO mice under these conditions (Figure 3, H and I, and Supplemental Figures 5 and 6).

### Leene-KO mice have impaired blood flow recovery after femoral artery ligation.

**Figure 4:**
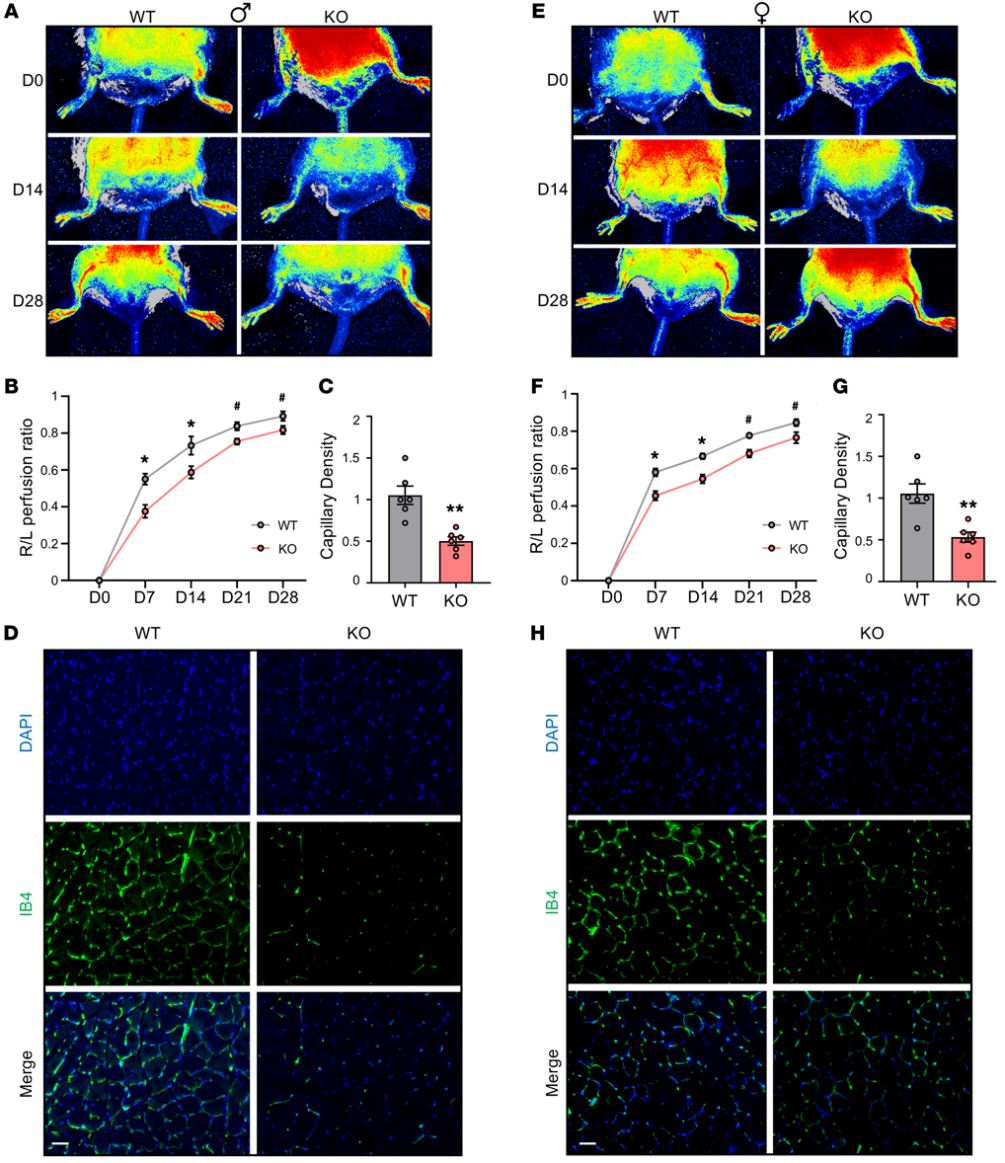
*Leene*-KO mice have impaired hind limb blood flow recovery after arterial ligation. Male (**A**–**D**) and female (**E**–**H**) mice were fed an HFHS diet for 16 weeks, followed by femoral artery ligation on the right hind limb and sham operation on the left on day 0 (D0). Perfusion recovery rate was measured at various time points (D0, D7, D14, D21, and D28) after femoral artery ligation by laser speckle flowgraphy. Data show blood perfusion ratio of the right to left (R/L) hind limb. Representative images (**A** and **E**) and quantitative analysis (**B** and **F**) (*n* = 8–10/group). (**C** and **G**) Quantification of capillary density based on IB4 staining (*n* = 6/group) and (**D** and **H**) representative images of IB4 (green) and DAPI (blue) staining in the gastrocnemius muscle collected 7 days after HLI. Scale bars: 50 μm. Data are represented as mean ± SEM. ^#^*P* = 0.05, *P < 0.05; **P < 0.01 based on 2-tailed Student’s *t* test.

To evaluate the effect of *Leene* deficiency in angiogenesis and ischemic response, we first subjected WT and KO mice to HLI under nondiabetic conditions. In KO animals fed normal chow, we observed a slight but insignificant trend toward a decrease in blood flow perfusion in response to ischemia, as compared with WT littermates (Supplemental Figure 7), suggesting other mechanisms may compensate for the loss of *Leene*. However, when challenged with an HFHS diet, which induces hyperglycemia and mimics a diabetic condition, the KO mice had significantly impaired flow recovery from HLI. Such decreased recovery was significant from postoperative day 7 and maintained up to day 28 (Figure 4, A and B), with lower microvascular density measured by IB4 staining (Figure 4, C and D). Similar phenotypes were observed in both male and female mice, with quantitative differences (Figure 4, E–H).

### LEENE RNA promotes ischemic recovery in vivo.

**Figure 5:**
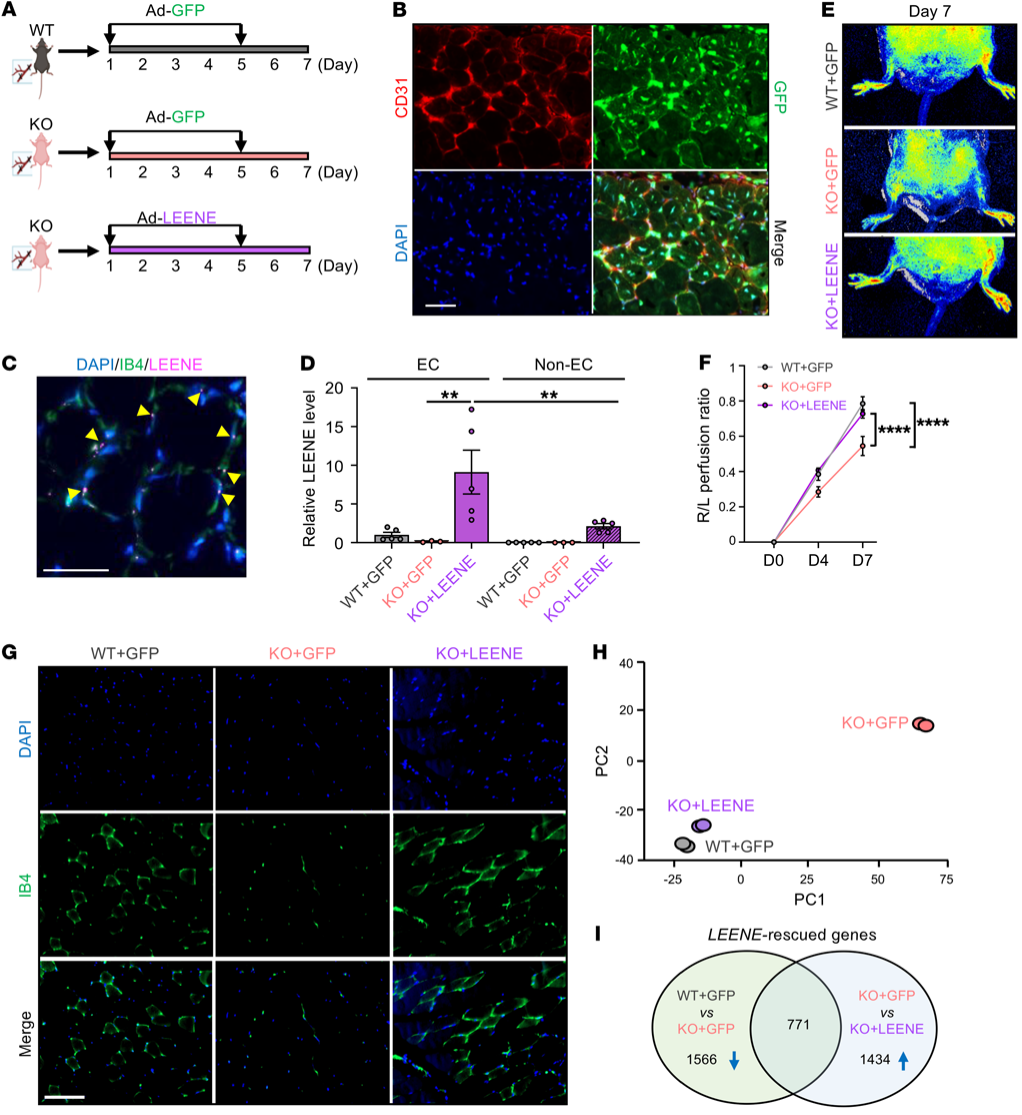
LEENE RNA promotes ischemic recovery in vivo. (**A**) Design of the rescue experiment: Adenovirus driving GFP (Ad-GFP) or LEENE (tagged by GFP) (Ad-LEENE) was injected intramuscularly on days 1 and 5 after HLI into WT or KO mice fed an HFHS diet. (**B** and **C**) Staining of CD31 and GFP (**B**) and smRNA FISH of LEENE and IB4 staining with DAPI counterstain (**C**) in the hind limb muscle of KO mice receiving Ad-LEENE. Arrows indicate colocalization of LEENE and IB4 signals. Scale bars: 50 μm. (**D**) qPCR of LEENE in EC-enriched and non–EC-enriched fractions isolated from the gastrocnemius muscle (*n* = 3–5/group). **P = 0.006 based on 1-way ANOVA followed by Tukey’s test. (**E** and **F**) Representative flowgraphy images (**E**) and quantitative analysis of perfusion recovery rate in the hind limbs (*n* = 4/group) (**F**). ****P < 0.0001 based on 2-way ANOVA followed by Tukey’s test. (**G**) IB4 staining in the hind limb muscle (*n* = 3–5/group). Scale bar: 100 μm. (**H**) PCA plot showing the gene expression of 3 groups profiled by RNA-seq with 2 replicates per group. (**I**) Venn diagram showing the LEENE-rescued genes, namely the overlap between downregulated by *Leene* KO (WT + GFP vs. KO + GFP) and upregulated by LEENE overexpression in KO (KO + GFP vs. KO + LEENE).

The observed phenotype in the KO mice could result from the deletion of *Leene* DNA and/or RNA. To delineate the contribution of LEENE RNA independent from its cognate DNA in the ischemic response and to test the function of human LEENE in vivo, we supplemented the HFHS diet–fed *Leene*-KO mice with human LEENE RNA. We injected an adenovirus expressing GFP-tagged human LEENE (Ad-LEENE) to the ischemic hind limb muscles of *Leene*-KO mice under the HFHS diet. As the vector control, adenovirus expressing GFP only (Ad-GFP) was injected into WT and KO mice (Figure 5A). The efficiency of adenoviral infection was confirmed by the positive GFP signal, which overlapped with CD31 (Figure 5B), as well as by the human LEENE signals detected by single-molecule RNA fluorescence in situ hybridization (smRNA FISH) in the KO mice, particularly in IB4-marked ECs (Figure 5C and Supplemental Figure 8). Overexpression (OE) of LEENE in the KO mice was also confirmed by qPCR, showing that the induction of LEENE was much higher in EC-enriched than in the non-EC fractions (Figure 5D).

**Figure 6:**
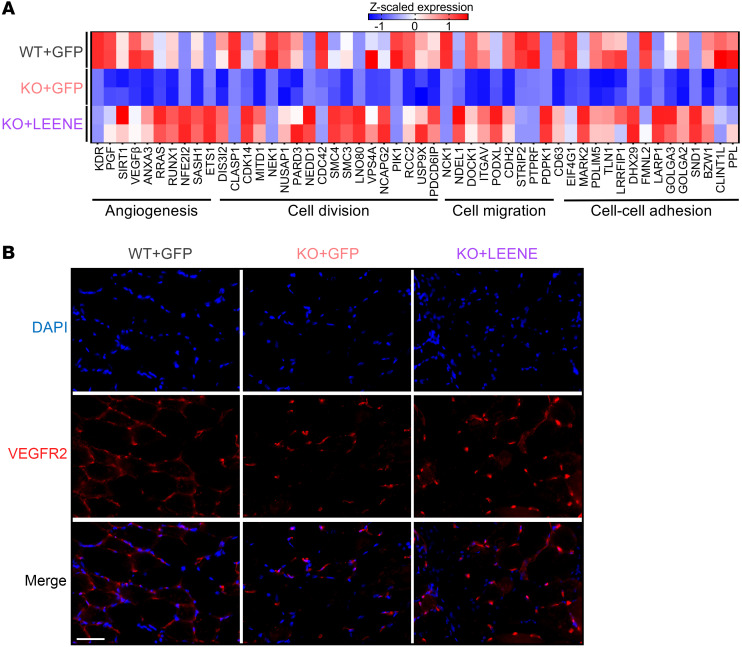
Human LEENE OE restores angiogenesis marker gene expression, including *KDR*. (**A**) Heatmap showing expression of select LEENE-rescued genes in EC-enriched fractions isolated from the gastrocnemius muscle, as described in Figure 5. (**B**) Immunofluorescent staining of VEGFR2 (encoded by *KDR*) with DAPI counterstain in the ischemic muscles. Scale bar: 50 μm. Data are represented as mean ± SEM.

While KO mice receiving control vector (KO + GFP) exhibited an impaired ischemic recovery compared with WT littermates receiving the same treatment (WT + GFP), Ad-LEENE injection in KO (KO + LEENE) led to a robust rescue of both perfusion and capillary density (Figure 5, E–G, and Supplemental Figure 9). Moreover, bulk RNA-seq of EC-enriched fractions isolated from ischemic hind limbs revealed that (a) the ischemic muscles from WT and KO mice had distinct gene expression profiles (by comparing WT + GFP vs. KO + GFP) and (b) LEENE OE in KO mice resulted in a transcriptome much more similar to that of WT (by comparing KO + GFP vs. KO + LEENE) (Figure 5H). Overlapping DEGs from these 2 comparisons identified 771 “LEENE-rescued” genes, which were downregulated by *Leene* KO but rescued by LEENE OE (Figure 5I). These genes were enriched for cell differentiation, division, migration, cell-cell adhesion, and angiogenesis, all important for ischemic response and tissue repair (Figure 6A and Supplemental Figure 10). As a candidate marker of angiogenesis, *KDR* was also in this list of genes, with its expression decreased by *Leene* KO and increased by LEENE OE, at both mRNA and protein levels (Figure 6 and Supplemental Figure 10). These data demonstrate that LEENE RNA, even in the absence of its cognate DNA, can promote angiogenesis and perfusion in response to ischemic injury especially under hyperglycemia.

### LEENE promotes angiogenic response in ECs and cell interactions to enhance ischemic recovery.

**Figure 7:**
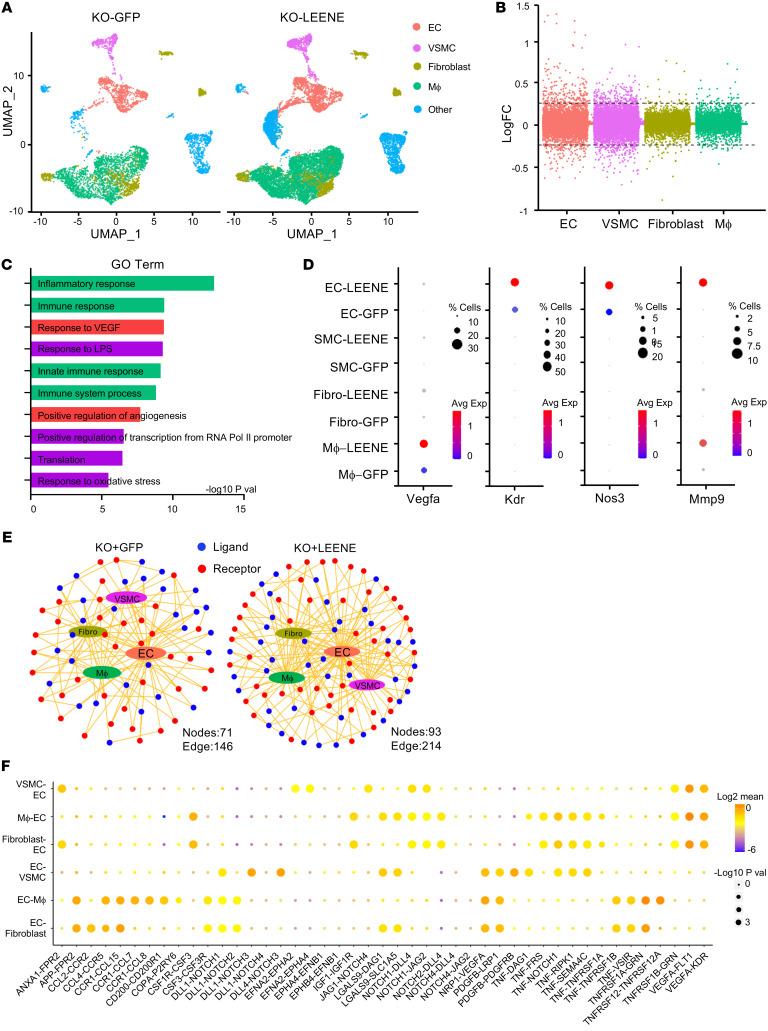
LEENE promotes angiogenic function in ECs and EC interactions with other vascular cells that promote ischemic recovery. *Leene*-KO mice were subjected to HLI and Ad-GFP/LEENE injection as illustrated in Figure 5A. Gastrocnemius muscles on the ischemic side were collected 7 days after HLI and the CD144-enriched fraction underwent scRNA-seq. Cells from 4 mice were pooled into 1 sample and 8 mice in total were used per group. Cells (5,573) from KO-GFP and 13,465 cells from KO-LEENE groups were profiled. (**A**) UMAP showing cell clusters identified from scRNA-seq split by condition (KO-GFP and KO-LEENE). (**B**) Manhattan plot showing logFC of all genes detected by scRNA-seq in different cell types. The cutoff for DEGs (log|FC| > 0.25) is indicated by the 2 horizontal dashed lines. (**C**) Top 10 enriched biological pathways of DEGs in ECs plotted with –log_10_(*P* value). (**D**) Dot plots showing representative gene expression in different cell types. (**E**) Network visualization of ligand (blue) and receptor (red) connectivity between ECs and VSMCs, macrophages (Mϕ), or fibroblasts (Fibro). Note the increase in the number of nodes and edges in the KO + LEENE group. (**F**) The increased, i.e., higher expression in KO + LEENE vs. KO + GFP or only detected in KO + LEENE but not in KO + GFP ligand-receptor interactions between ECs and other cell types in the hind limb by LEENE OE. The size of spheres indicates –log_10_(*P* value) between the 2 groups and color indicates expression of the ligand-receptor pair in the corresponding cell types.

The ischemic response engages a multitude of cells in addition to MVECs, such as vascular smooth muscle cells (VSMCs), immune cells, and fibroblasts (26–29). To dissect the effect of LEENE OE in ECs and other relevant cell types, we performed scRNA-seq with EC-enriched cell populations isolated from ischemic muscles of KO mice receiving Ad-GFP or Ad-LEENE. Among approximately 20,000 cells sequenced, approximately 3,000 were ECs, approximately 1,000 VSMCs, approximately 5,700 monocytes/macrophages, and approximately 5,200 fibroblasts (Figure 7A). DEG analysis identified 276 DEGs in ECs (172 up- and 104 downregulated by Ad-LEENE) (Figure 7B), which were enriched for inflammatory and immune responses, as well as angiogenesis (Figure 7C). In VSMCs, scRNA-seq identified 330 DEGs that were enriched in translation and several pathways related to tissue regeneration, e.g., organ regeneration and wound healing (Supplemental Figure 11). In contrast, in monocytes and fibroblasts, the number of DEGs was much lower (i.e., <50 each) (Figure 7B).

scRNA-seq also detected key marker genes for ischemic response with cell specificity. For example, *Vegfa* was detected and induced by LEENE OE in multiple cell types, with highest expression in macrophages. *Kdr* and *Nos3* were predominantly expressed in ECs with increased expression by LEENE OE. *Mmp9*, encoding matrix metalloproteinase 9 that has been shown to be essential for ischemia-induced neovascularization (30), was detected and induced by LEENE in both ECs and macrophages (Figure 7D). We also examined the cell interactions using CellPhoneDB, which infers cell-cell communication based on the expression of ligand-receptor pairs (31). LEENE OE led to more active EC crosstalk with other cell types, as indicated by more nodes and edges in ligand-receptor interactions radiating from ECs (Figure 7E and Supplemental Figure 12). Among these interactions, a large proportion was involved in angiogenic pathways, including VEGF, PDGF, and Notch signaling (Figure 7F). Taken together, these results suggest that LEENE RNA promotes ischemic recovery through enhancing EC angiogenic function as well as EC interactions with other vascular cells.

### Interaction of LEENE with genome, LEO1, and MYC promotes the transcription of angiogenic genes.

**Figure 8:**
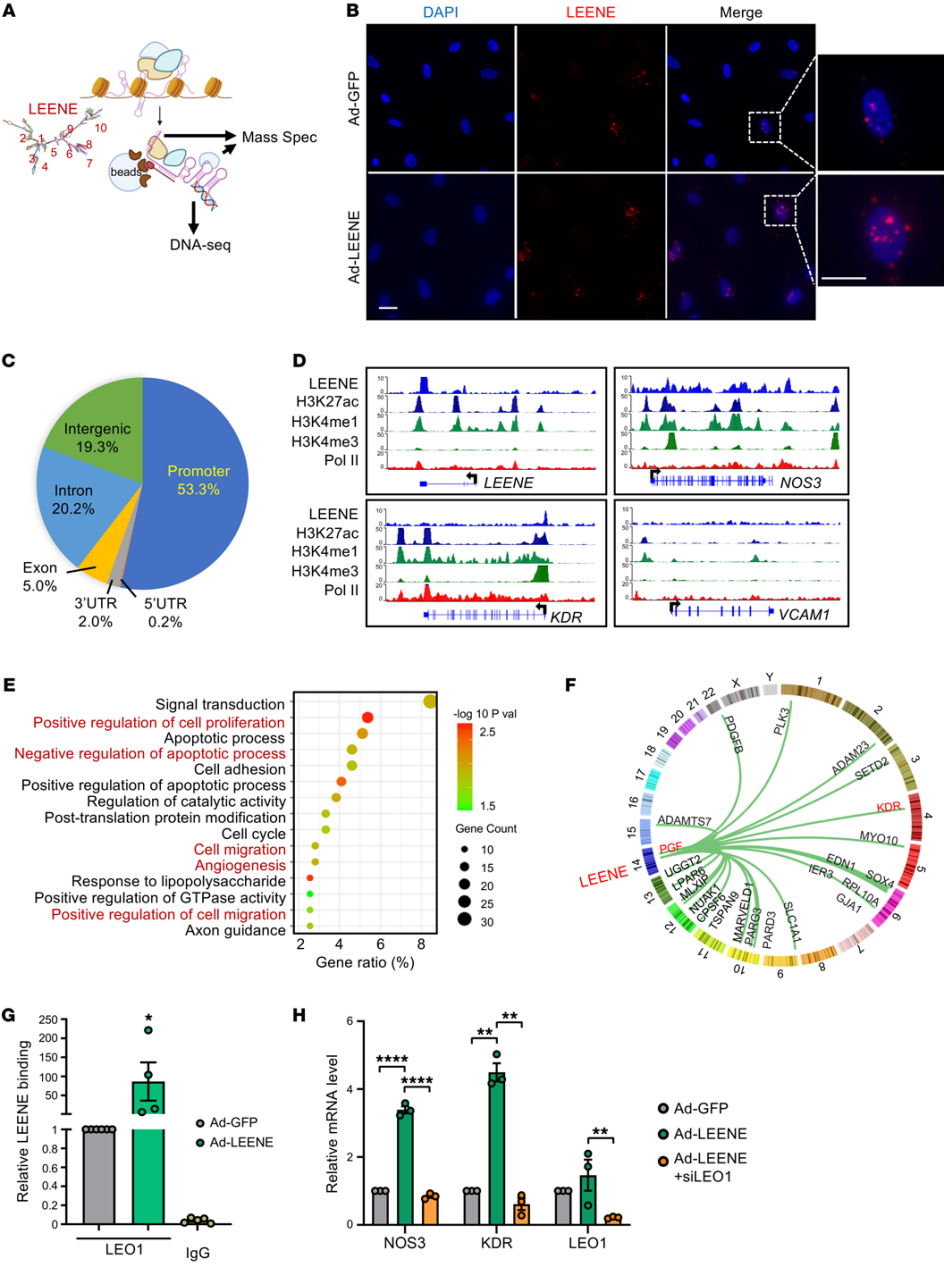
LEENE interacts with promoters and LEO1 to increase proangiogenic gene expression. (**A**) Schematic diagram showing ChIRP performed with 10 biotinylated probes, the locations of which are shown based on the predicted secondary structure of LEENE. The ChIRP precipitates were subjected to DNA-seq and mass spectrometry. (**B**) smRNA FISH of LEENE in ECs infected with Ad-GFP or Ad-LEENE. Scale bar: 50 μm. (**C** and **D**) ChIRP-seq was performed with Ad-LEENE–infected HUVECs in biological triplicates. (**C**) Pie chart showing the proportion of reads aligned to different genomic regions. (**D**) LEENE binding signals in representative genes (top blue tracks) in parallel to HUVEC ChIP-seq data from ENCODE. (**E**) GO analysis of top 15 enriched pathways in LEENE interactome and regulome, i.e., 395 genes downregulated by LEENE KD in vitro and showing genomic interaction with LEENE. (**F**) Circle plot showing LEENE interaction with 23 LEENE-regulated (in vitro and in vivo) and -interacting genes. (**G**) RIP performed with HMVECs and anti-LEO1 antibody with IgG as an isotype control. LEENE RNA in the immunoprecipitates was quantified by qPCR and the relative enrichment in the Ad-GFP sample was set to 1. *P = 0.04 based on 1-way ANOVA followed by Dunnett’s test. (**H**) qPCR analysis of *NOS3*, *KDR*, and *LEO1* in HMVECs transfected by scramble or LEO1 siRNA (siLEO1) and infected by Ad-GFP or Ad-LEENE. Bar graphs represent mean ± SEM. **P < 0.01; ****P < 0.0001 compared with Ad-GFP or between indicated groups based on 1-way ANOVA followed by Dunnett’s test.

We explored the potential mechanism underlying LEENE-promoted angiogenesis. Given the nuclear localization and chromatin-binding feature of LEENE (18), and its effect on the transcriptome, we reasoned that LEENE plays a role in transcriptional regulation. To gain a comprehensive view of the LEENE-chromatin interactome, we performed chromatin isolation with RNA pulldown (ChIRP) to pull down LEENE-associated DNA and proteins. Specifically, we used 10 previously validated tiling nucleotides targeting different domains based on the predicted secondary structure of LEENE (18) (Figure 8A). On one hand, we performed ChIRP followed by DNA-seq to map LEENE-interacting genomic regions. To enhance the ChIRP-seq signals, we overexpressed LEENE in ECs and confirmed the nuclear and chromatin enrichment of LEENE (Figure 8B and Supplemental Figure 13). ChIRP-seq performed in 3 replicates revealed that over 50% of LEENE-bound regions resided in promoter proximity (TSS ± 3 kb). The rest were in introns (20.2%), intergenic regions (19.3%), exons (5%), etc. (Figure 8C). For example, LEENE RNA bound to its own and *NOS3* DNA loci as expected (18); LEENE also bound to the *KDR* promoter (Figure 8D). In contrast, no LEENE binding was detected in *VCAM1*, which is not directly regulated by LEENE (18) (Figure 8D). These binding signals were verified by ChIRP-qPCR using ECs with endogenous LEENE expression, whereas the control LacZ probes did not yield significant signals (Supplemental Figure 14). Integrating ChIRP-seq with the RNA-seq data from ECs with LEENE KD, we identified 395 genes whose expression was positively regulated by LEENE and showed binding in their genomic loci (Supplemental Figure 15). Many of these genes were involved in angiogenesis, cell proliferation, and cell migration (Figure 8E). To identify putative target genes positively regulated by LEENE through binding, we analyzed for common genes that are consistently and positively regulated by LEENE/Leene in vitro and in vivo and identified 23 genes that may be regulated by LEENE through binding in its DNA (Supplemental Figure 16). These target genes include *KDR*, *PGF*, and *PDGFB*, encoding a well-characterized receptor and growth factors that drive angiogenesis (Figure 8F).

On the other hand, we performed ChIRP–mass spectrometry (ChIRP-MS) to identify LEENE-interacting proteins that may mediate the LEENE-regulated transcription. ChIRP-MS detected 15 candidate proteins that consistently appeared in ECs with endogenous and exogenous LEENE and were unlikely to be common contaminants (Supplemental Figure 17). Among these, we found LEO1 of particular interest given its reported role as a key subunit of RNA polymerase II–associated factor complex (Paf1C) and in gene transcription and chromatin states (32–34). Western blotting using a validated anti-LEO1 antibody (35, 36) confirmed the association of LEO1 with LEENE in ECs with endogenous or exogenous LEENE. This could be also achieved by splitting the 10 probes into odd- and even-numbered probes, but not when the samples were treated with RNase A (Supplemental Figure 18), indicating the pulldown of LEO1 is RNA dependent. Reciprocally, ribonucleoprotein immunoprecipitation (RIP) using UV-crosslinked EC nuclear extracts showed that LEO1 IP enriched LEENE, which was not observed with IgG (Figure 8G). To test whether LEO1 is involved in LEENE-regulated angiogenic gene expression, we chose *NOS3* and *KDR* as prototypic candidates given their key functions in ECs. When LEO1 was knocked down in HMVECs, the induction of *NOS3* and *KDR* by LEENE OE was abolished, supporting a requirement of LEO1 in LEENE’s induction of *NOS3* and *KDR* (Figure 8H).

**Figure 9:**
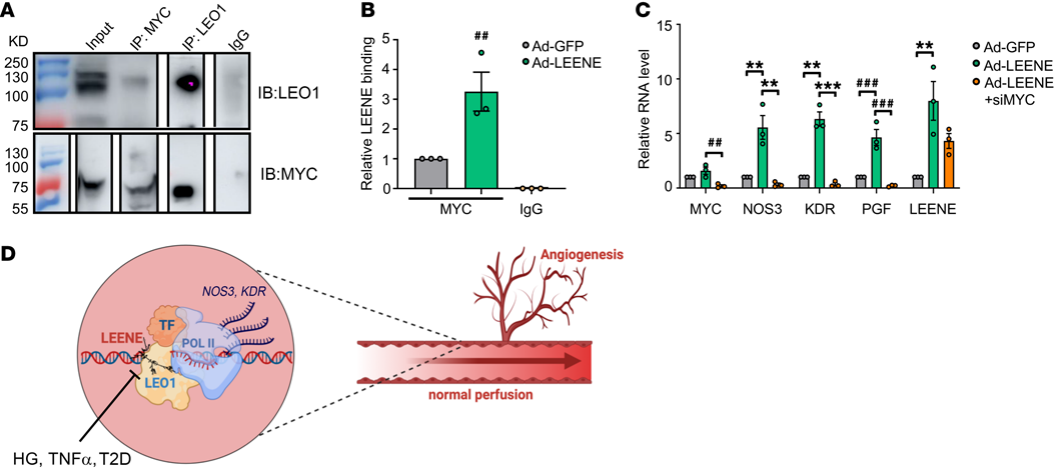
Involvement of MYC in the LEENE-LEO1 mechanism. (**A**) Co-IP of LEO1 and MYC in ECs. (**B**) RIP with ECs infected with Ad-GFP or Ad-LEENE using anti-MYC antibody or IgG control. LEENE RNA in the immunoprecipitates was quantified by qPCR and the relative enrichment in the Ad-GFP sample was set to 1. (**C**) qPCR analysis of ECs transfected with scramble or MYC siRNA (siMYC) and infected with Ad-GFP or Ad-LEENE. Bar graphs represent mean ± SEM. ^##^*P* = 0.01; **P < 0.01; ^###^*P* = 0.001; ***P < 0.001 based on 1-way ANOVA followed by Dunnett’s test. (**D**) Schematic illustration of LEENE-regulated angiogenic and ischemic responses. LEENE, potentially by binding LEO1 and MYC, promotes the transcription of proangiogenic genes, e.g., those encoding eNOS (*NOS3*) and VEGFR2 (*KDR*), to enhance angiogenesis and flow perfusion. Such mechanism is suppressed in diabetic conditions, which contributes to the reduced tissue perfusion in PAD.

To identify the potential mechanism underlying how LEENE guides LEO1 protein to regulate the proposed target genes, we further explored the potential role of key TFs, which can direct specificity of target transcriptional activation. To this end, we selected MYC given the following reasons: (a) MYC interacts with LEO1 in *Drosophila* and this contributes to the transcriptional induction of MYC’s targets (37); (b) MYC has been shown to be essential for angiogenesis and positively regulates *KDR* and *PGF* (38, 39), which are also induced by LEENE; MYC is also among the putative TFs involved in the LEENE regulome based on IPA; and (c) MYC has been shown to bind to lncRNA, which leads to its enhanced transactivation activity, using 2 commonly used RNA-binding-protein prediction tools, RNAct and RPIseq (40, 41). MYC was found to be a highly-ranked (no. 3) putative binding protein of LEENE (Supplemental Table 5). IP of LEO1 pulled down MYC protein; reciprocally, IP of MYC pulled down LEO1 (Figure 9A). We then performed RIP with an anti-MYC antibody using extracts from ECs. As compared with the IgG control, anti-MYC antibody captured significantly more LEENE RNA from ECs. When LEENE was overexpressed using Ad-LEENE, the LEENE RNA pulled down using anti-MYC antibody was further increased, suggesting that MYC binds to LEENE (Figure 9B). Furthermore, MYC KD abrogated LEENE’s induction of *NOS3*, *KDR*, and *PGF*, without significantly reducing LEENE (Figure 9C). Taken together, our data suggest LEENE may promote the angiogenic gene transcription through association with TFs (e.g., MYC) and LEO1.

## Discussion

In the present study, we identified LEENE as an essential lncRNA in governing angiogenesis and tissue perfusion. Inhibition of LEENE in human ECs or deletion of the *Leene* homolog in mice, mimicking the effect of diabetic conditions, impairs angiogenic capacity with downregulation of genes that promote angiogenesis. Importantly, in *Leene*-KO mice, the impaired blood flow recovery after femoral artery ligation in diabetic mice was restored by human LEENE OE, evident at tissue function and single-cell transcriptome levels. Lastly, LEENE RNA engages LEO1 and MYC protein to promote the transcription of key genes in angiogenesis, e.g., *NOS3* and *KDR*. Collectively, these findings demonstrate a crucial role of LEENE in regulating angiogenesis and ischemic recovery (Figure 9D).

The reduced angiogenic capacity of HMVECs by LEENE KD and impaired hind limb flow in *Leene*-KO mice consistently support a positive role of LEENE in angiogenesis and ischemic recovery. To corroborate this, inhibition of LEENE RNA or deletion of *Leene* at the DNA level led to profound suppression of an angiogenic gene program. Comparison between the DEGs by LEENE KD in vitro and those by *Leene*-KO in vivo revealed 58 genes consistently downregulated in vivo, including several key players in angiogenesis, e.g., *KDR*, *PGF*, and and *PDGFB* (Supplemental Figure 19). We created the *Leene*-KO mice by deleting the mouse syntenic region of human LEENE to investigate the functional importance of this gene in vivo. After we established the KO mice, 2 other transcripts, *Gm35360* (a lncRNA divergently transcribed immediately upstream of *Leene*/*Gm41148*) and *Gm49302* (a lncRNA embedded in the intron of *Leene*) were annotated in the same region. Based on the Molecular Evolutionary Genetics Analysis (42) and sequence alignment, Gm41148 shows the highest homology to human LEENE (Supplemental Figure 20). Although the expression levels of these lncRNAs appeared to be lower than that of *Leene* (Supplemental Figure 21), we cannot exclude the possibility that their deletion may also contribute to the observed phenotype of the KO mice. Nevertheless, the robust rescue of LEENE in the KO mice provides strong evidence for a positive role of LEENE RNA in angiogenesis and ischemic recovery in vivo. The profound effect of LEENE OE to induce a transcriptomic profile in KO mice that resembled that of WT mice also demonstrates a function of LEENE RNA in gene regulation independent of its DNA. However, overexpression could restore transcript levels beyond physiological set points and thus may not fully recapitulate the effect of endogenous LEENE RNA. Future studies are warranted to further examine the role of endogenous LEENE RNA in physiological conditions in vivo.

We focused on the role of LEENE in ECs given that ECs are the major effector cell of angiogenesis, perfusion, and ischemic recovery. It is possible that *Leene* deletion in other cell types participates in this process. Nonetheless, the relatively high expression of Leene in EC-enriched compared with non–EC-enriched fractions suggests that ECs are a primary cell type in which Leene functions. By examining publicly available databases, including GTEX, FANTOM, and ENCODE, and other available data sets in the NCBI Gene Expression Omnibus (GEO), we observed that LEENE is broadly expressed in various tissues and organs. However, compared with several other relevant cell types, including VSMCs, monocytes, fibroblasts, and skeletal myocytes, LEENE seems more active in ECs, as indicated by H3K27ac ChIP-seq and RNA-seq data (Supplemental Figure 22). Furthermore, the OE of human LEENE in murine capillary ECs in vivo, the considerably higher induction of LEENE in EC-enriched fractions, and consequently the upregulation of EC-specific genes, e.g., *Nos3* and *Kdr* in the HLI model, also support ECs as one of the primary cell types where LEENE exerts its effect. However, we cannot exclude the potential role of LEENE in other cell types and their differential effects in diluting the EC effect. For example, Ad-LEENE also resulted in changes in 330 DEGs involved in organ regeneration and wound healing in VSMCs, likely through both cell-autonomous and paracrine effects. It would be of interest to explore in future studies the regulatory role of LEENE in other vascular cells.

Mechanistically, LEENE may regulate transcription in *trans*. Human LEENE is not coexpressed with the 2 neighboring genes *KTN1* and *PELI2* in ECs (18) and neither KD nor OE of LEENE significantly affects these genes (Supplemental Figure 23). In *Leene*-KO mice, neither *Ktn1* nor *Peli2* showed a marked change as compared with WT littermates (Supplemental Figure 23). Thus, we did not focus on the potential *cis* effect of *LEENE*/*Leene* in the current study. In contrast, gain or loss of function of LEENE/Leene resulted in extensive changes in expression of genes involved in angiogenesis, e.g., *NOS3*, *KDR*, and *PGF*, which are distantly encoded from *LEENE* but showed genomic binding by LEENE RNA. In diabetic conditions, a number of lncRNAs have been shown to regulate target genes associated with inflammation and diabetic complications by interactions with key RNA-binding proteins (6). In search of potential LEENE-binding proteins, we identified LEO1, the silencing of which abolished LEENE’s induction of *KDR* and *NOS3*. LEO1 is a highly conserved RNA-binding protein required for Paf1C interaction with nascent RNA and the trimethylation of H3K4, a marker of promoters (43) within transcribed chromatin (6, 32). However, LEO1 and Paf1C have mostly been studied in lower eukaryotic organisms (34, 43). LEO1 loss of function causes cardiac defects in zebrafish (44), implying a role of LEO1 in the cardiovascular system. In line with the reported role of LEO1 in mRNA transcription and histone modification, LEENE OE increased the nascent RNA synthesis of *KDR* and *PGF* with an attendant increase in H3K4me3 in their promoters (Supplemental Figure 24). Additionally, we showed that LEENE associates with MYC, a key TF in angiogenesis that has been shown to interact with LEO1 (37). This likely provides a basis for specificity for the proangiogenic genes. Upstream regulator analysis of the LEENE interactome and regulome (i.e., 395 genes downregulated by LEENE KD and showing LEENE-genome interactions) predicts 2 additional interesting TFs involved in the LEENE-regulated transcriptome network. One is KLF2, a key TF maintaining EC identity and homeostasis (45) and the other HIF1α, the pivotal TF activated by hypoxia to promote angiogenesis (Supplemental Figure 25 and Supplemental Table 6). Moreover, the putative binding sequences for KLF4 and HIF1α were enriched in the LEENE-bound DNA sequences profiled by ChIRP-seq (Supplemental Figure 25). Of note, LEENE itself, with multiple putative binding sites in its promoter, can also be transcriptionally induced by KLF2/4 and HIF1α (18) (Figure 1C and Supplemental Figure 25). Knockdown of KLF2 also abolished LEENE’s induction of *NOS3*, *KDR*, and *PGF* (Supplemental Figure 26). Taken together, our data suggest that LEENE functions through interactions with TFs and LEO1 to promote the transcriptional activation of angiogenesis. Future work will be needed to further investigate the stoichiometric basis for LEENE activities and its regulatory mechanism with these TFs.

Aberrant angiogenesis and EC dysfunction are key features associated with impaired tissue perfusion and repair in diabetes. In the context of PAD, multiple mechanisms have been identified, including defective expression or signaling of angiogenic molecules, impaired mobilization of endothelial progenitor cells, and inflammation that selectively promotes tissue damage over repair (46). Although most of these processes have been targeted to treat PAD, there has been no clinical success to date. Thus, novel strategies are needed to address this unmet medical need. The consistent suppression of LEENE/Leene in human and murine ECs exposed to diabetic conditions and in human arteries from (pre)diabetic donors suggests that the dysregulation of LEENE may be a major mechanism underlying EC dysfunction, early in diabetes, contributing to long-term vascular damage (47). Accumulating studies suggest that failure of angiogenic strategies in PAD is likely due to impaired downstream signaling pathways (46). Furthermore, in PAD patients, impaired eNOS-NO bioavailability is another causal mechanism that may not be restored by proangiogenic therapy. Thus, the dual effect of LEENE in promoting the expression both eNOS and other proangiogenic genes, including that of growth factors (e.g., PGF and PDGF) and receptors (e.g., VEGFR2), would be highly desirable for ameliorating EC dysfunction and improving tissue perfusion in PAD. Additionally, although there was no apparent defect in ischemic response in *Leene*-KO mice, the retinal angiogenesis during development was compromised in the KO mice (Supplemental Figure 27), suggesting that LEENE-mediated angiogenesis is an essential process during development as well as diabetes-associated lower limb ischemia. As such, our studies provide a proof of concept for a lncRNA-based strategy to improve tissue repair and regeneration by restoring the EC functional transcriptome via epigenetic mechanisms. Our studies suggest that therapeutic manipulation of LEENE may complement existing therapeutic strategies for the management of ischemic diseases and diabetic vasculopathies.

## Methods

For extended methods, please refer to the supplemental materials.

### Generation of Leene-KO mice.

*Leene*-KO mice were generated at the City of Hope Transgenic Mouse Facility by deleting the syntenic region of human LEENE in the mouse genome (illustrated in Supplemental Figure 4). Specifically, an approximately 47 kb region (mm39 chr14: 48,019,230–48,066,441) was targeted using CRISPR/Cas9 gene editing. The CRISPR single guide RNAs (sgRNAs) were designed to have the lowest off-targeting potential using the CHOPCHOP CRISPR design program (48), synthesized (Synthego, Inc.), and tested by using the IDT Surveyor Mutation Detection Kit. The sgRNAs with the highest cutting efficiency (sequences provided in Supplemental Table 7) were selected and complexed with 50 ng/μL AltR Cas9 protein (IDT, Inc.) at a final concentration of 25 ng/μL for 10 minutes at 37°C. The mixture was then microinjected into fertilized C57BL/6J 1-cell embryos, which were then implanted into pseudopregnant recipient female mice. Genotypes were determined with different primers by PCR to detect the intact *Leene* locus in WT or its deletion in KO mice. The F0 homozygous KO mice were crossed with WT C57BL/6J mice (The Jackson Laboratory) for 5 more generations before further experimentation. Both male and female mice were used in this study.

### Murine diabetes model and metabolic phenotyping.

To induce obesity and hyperglycemia in mice and characterize the metabolic phenotype, we followed a published protocol (21). Briefly, mice were randomized to receive irradiated HFHS diet (17% kcal protein, 32% kcal fat, 51% kcal carbohydrate; D12266B, Research Diets Inc.) starting at 8 weeks old for 16 weeks. Mice receiving a regular chow diet (D12489B, Research Diets Inc.) were kept for the same duration. Body weight was measured at the initiation of the HFHS diet and subsequently every other week. Body composition measurement and glucose tolerance test (GTT) were performed by the Comprehensive Metabolic Phenotyping Core of City of Hope. Body composition was measured using magnetic resonance imaging (MRI, EchoMRI). GTT was performed with mice fasted for 5 hours with free access to water. Glucose (0.3 g/mL) was injected into the peritoneal cavity based on the body weight (1.5 g glucose/kg body weight). Tail vein blood was drawn at 15, 30, 60, 90, and 120 minutes after the injection for blood glucose measurement using a FreeStyle Freedom Lite glucometer (Abbot Diabetes Care, Inc.). The area under curve (AUC) was compared between genotypes.

### HLI model and adenoviral injection.

HLI was performed as described previously (26) with modifications. The surgical site was shaved and treated with topical antiseptic. The left femoral artery was isolated and occluded 5–6 mm distal to the inguinal ligament by ligation with 6-0 surgical silk. The control limb on the right side underwent the same without arterial ligation. Blood perfusion was measured by laser speckle flowgraphy using a PERICAM PSI Z system (Perimed AB). The ratio of blood flow in the ischemic to nonischemic hind limb was calculated and expressed as the perfusion recovery rate. Adenovirus injection was performed following a previously described method (49). Purified adenovirus particles were injected at 3 sites in the adductor muscle and 1 site in the gastrocnemius muscle of the ischemic hind limb on days 1 and 5 after HLI surgery. At the endpoints, mice were euthanized by CO_2_ inhalation.

### Sequencing data analysis.

RNA-seq data were analyzed as described previously (19, 21). Briefly, for RNA-seq, STAR (50) was used to align raw sequencing data to the hg38 or mm9 genome and Kallisto (51) was used to quantify transcript abundance in transcripts per million (TPM) values. DESeq2 (52) was then used to perform DEG analysis with default parameters (adjusted *P* values < 0.05 were considered significant). GO pathway enrichment analysis was performed through the Gene Ontology Consortium Platform 55 (https://david.ncifcrf.gov/) and Benjamini-Hochberg–corrected *P* values of less than 0.05 were considered to indicate significantly enriched pathways.

To identify candidate lncRNAs involved in impaired angiogenesis in diabetic conditions, 3 sets of RNA-seq data from ECs under 6 different treatments, i.e., HG versus normal glucose osmolarity control (NG), TNF-α versus nontreated control, and hypoxia versus normoxia were used. The expression data sets were filtered for lncRNAs that are (a) differentially expressed in the diabetic conditions (HG and/or TNF-α) and proangiogenic hypoxia, as compared to their respective control; and (b) changed in opposite pattern by diabetic versus proangiogenic conditions. This generated 2 groups of lncRNAs shown in Figure 1A, i.e., HG- and TNF-α–upregulated and hypoxia-downregulated and HG- and TNF-α–downregulated and hypoxia-upregulated, in a total of 92 lncRNA candidates. Subsequently, we ordered these lncRNAs by the extent of changes due to HG and TNF-α as compared with hypoxia. We used the following equation to calculate the extent of changes for ranking: |FC(Hx vs. Nx) – FC(HG vs. NG)| + |FC(Hx vs. Nx) – FC(TNF-α vs. control)|, where Hx and Nx are hypoxia and normoxia.

ChIRP-seq was analyzed following a published pipeline (53). Reads from 3 biological replicates were aligned to human genome hg38 using Bowtie (54). Only uniquely aligned reads were used for the subsequent peak calling using MACS2 (55) against its corresponding input with a cutoff of *P* less than 1 × 10^–5^. LEENE target reads were annotated using Ensembl GRCh38.86 as reference. Annotation was performed against gene bodies using 1-base overlap. ChIP-seq data from HUVECs in biological replicates were analyzed as previously described (56). Briefly, the deduplicated alignments were subjected to peak calling by MACS2 and annotated by the Homer suite (57). Loci with ChIP-seq signals were identified by bedtools and visualized by WashU Epigenome Browser (https://epigenomegateway.wustl.edu/).

scRNA-seq data were processed using a standardized pipeline provided by 10× Genomics (v6.0.1) and aligned to mouse mm10 reference transcriptome (v3.0.0). The R package Seurat (v3.2.2) was used to analyze scRNA-seq data following published guidelines (58). First, we performed a filtering step using well-established quality control metrics. Rare cells with over 3,000 expressed genes (potentially multiplets), as well as mitochondrial percentage over 25% (indicating low-quality or dying cells) were removed. Next, data were normalized using “sctransform” to improve sample integration. The normalized data were used for dimensionality reduction and clustering (we used the first 15 principal components and resolution = 0.5 as a clustering parameter), while log-normalized expression levels were used for analysis based on gene expression level, including cell type classification and differential expression analysis. Based on the literature (26, 59), we selected the following marker genes: *Cdh5* and *Pecam1* for ECs, *Acta2* for VSMCs, *Itgam*, *Adgre1*, and *Fcgr1* for macrophages, and *S100a4* for fibroblasts. Thus, we first computed the average expression level across single cells between each set of markers in order to have a single marker with an average expression level associated with each cell type. Then, we applied a Gaussian mixture model with 2 components to the expression data of each marker across single cells to separate cells into 2 sets, namely those expressing high levels of the markers and those expressing low levels. For each marker, each single cell was assigned to 1 of the 2 components. Finally, we used statistical enrichment for the set of marker genes and a Fisher’s exact test to assign a cell type to each cluster. By doing so we obtained a set of *P* values for each cluster, each corresponding to a cell type. The cell type with the lowest *P* value was assigned to that cluster. Differential expression analysis was performed using Seurat with default parameters.

Cell-cell communication analysis was performed using the publicly available CellPhoneDB (31) with default parameters. To use the tool on mouse data, we converted the mouse genes to human homologs using the conversion table available at the Mouse Genome Database (60). Cell-cell communication networks were plotted using Cytoscape v3.5.1 (https://cytoscape.org/).

### Data availability.

RNA-seq data from HUVECs treated with HG and scRNA-seq data from human mesenteric arteries were previously published (19) and are available at the NCBI GEO with accession no. GSE135357. RNA-seq data from HUVECs and Human Aortic Endothelial Cells (HAECs) treated with TNF-α and HMVECs treated with hypoxia were previously published (20, 21) and are available at GEO with accession nos. GSE163433 and GSE136912. ChIP-seq data from HUVECs treated with HG and TNF-α, bulk RNA-seq from HUVECs with LEENE KD, bulk RNA-seq and scRNA-seq data from mouse experiments, and ChIRP-seq data from HUVECs have been uploaded to GEO with accession number GSE205287. ChIRP-MS data have been uploaded to MassIVE with identifier no. MSV000089412.

### Statistics.

Statistical analyses for data other than high-throughput sequencing were performed using GraphPad Prism. Two-group comparisons were performed using 2-sided Student’s *t* test and multiple-group comparisons were performed using ANOVA followed by post hoc test as appropriate (specified in figure legends). *P* values less than 0.05 were considered statistically significant.

### Study approval.

Animal experiments were approved by the Institutional Animal Care and Use Committees at City of Hope. Human tissue studies were conducted on deidentified specimens obtained from the Southern California Islet Cell Resource Center at City of Hope. Research consents for the use of postmortem human tissues were obtained from the donor next of kin and ethical approval was granted by the Institutional Review Board of City of Hope (IRB 01046). Nondiabetic control donors were selected based on available medical records, which showed glycated hemoglobin A1c (HbA1c) within the normal level (<5.7%) and the lack of any known metabolic and cardiovascular conditions. Severe obesity was defined as a body mass index (BMI) above 35 but under 40 and morbid obesity was defined as a BMI of 40 or greater. Pre-T2D and T2D were identified based on the donors’ medical records and HbA1c level between 5.7% and 6.4% (for pre-T2D) or 6.5% or higher (for diabetes) (Supplemental Table 2).

## Author contributions

RN, JPC, SZ, and ZBC conceived and designed the experiments. XT and YM designed and conducted most of the in vivo studies and some of the in vitro experiments. YL, DY, NKM, and KS designed and conducted the in vitro experiments and analyses. YL and DY generated all the high-throughput sequencing libraries, and DY, RC, AT, and ATC performed the subsequent analysis. CHL, WT, and ZBC designed and generated the KO mice. XT and YL validated the KO mice. DR performed the mass spectrometry experiment and data analysis and MK supervised these experiments and interpreted results. GZ performed cardiac function assessment and ZVW supervised these experiments. XT and ZBC wrote the original draft of the manuscript. YL, DY, JPC, RN, SC, MK, and ZVW revised the manuscript and provided critical discussion. SZ, SC, RN, MK, and ZBC obtained funding for this study. XT, YL, and DY are assigned co–first authors in an order mainly based on their contribution in generating data supporting the major conclusion.

## Supplementary Material

Supplemental data

## Version 1 12/13/2022

In-Press Preview

## Version 2 02/01/2023

Electronic publication

## Version 3 07/11/2024

Corrected 3 text errors
