# Supplementary material for: Long noncoding RNA LEENE promotes angiogenesis and ischemic recovery in diabetes models
Source: J Clin Invest. 2022 Dec 13;133(3):e161759. doi: 10.1172/JCI161759 (PMC9888385; doi:10.1172/JCI161759)
Supplement: Supplemental data [file jci-133-161759-s010.pdf]

SUPPLEMENTAL MATERIAL

Supplemental Methods

Metabolic phenotyping

Body composition measurement and glucose tolerance test (GTT) were performed by Comprehensive Metabolic Phenotyping Core (CMP) of City of Hope. Body composition was measured using magnetic resonance imaging (MRI, EchoMRI, Houston, TX). Each mouse was scanned for 1-2 min without anesthesia. The scores representing the total fat and total lean mass in grams were further calculated to compare the difference between different genotypes.

Mice receiving GTT were fasted for 5 hours prior to the procedure, with free access to water. Glucose (0.3 g/ml) or insulin (0.5 IU insulin/kg body weight, Humulin R U-100, Lilly) was injected into the peritoneal cavity based on the body weight (1.5 g glucose/kg body weight). Tail vein blood was drawn at 15, 30, 60, 90, and 120 mins after the injection for blood glucose measurement by using a FreeStyle Freedom Lite glucometer (Abbot Diabetes Care, Inc., Alameda, CA).

Isolation of ECs from murine lungs and hindlimb muscles, and intima from human mesenteric arteries

Murine lung ECs were isolated as previously described (1). Lungs were digested with Type I collagenase (Worthington Biochemical). Similarly, ECs were isolated from gastrocnemius muscles with a digestion buffer containing Type I collagenase in DMEM (1mg/ml). Sorting was done with anti-CD144-conjugated magnetic beads and MACS columns (Miltenyi Biotec). The

intimal RNA was isolated from human mesenteric artery by flushing once the inner lumen of mesenteric arteries with TRIzol as published (2).

Cell culture, transfection, and treatment

HMVECs and HUVECs were purchased from and verified for negativity for mycoplasma contamination by Cell Applications, Inc. EC identity was authenticated by using immunostaining, flow cytometry, and the expression of CD144, CD31, and eNOS mRNA. HMVECs at passages 4-6 were cultured in HMVEC growth medium and HUVECs at passages 5–7 were cultured in M199 medium supplemented with growth factors, heparin sodium under standard cell culture conditions (humidified atmosphere, 5% CO_2, 37°C) (1). HG condition was generated by adding D-glucose into the culture media to a final concentration of 25 mM. As normal glucose/osmolarity control, mannitol was added at 20 mM to the cells cultured in medium with 5 mM glucose. TNFα was added to the culture media to a final concentration of 100 ng/mL or 5 ng/mL for a combined HG and TNFα treatment (HT) for H3K27ac ChIP. The normoxic cells were kept at 37°C ventilated with 5% CO_2 and atmospheric 21% oxygen. The hypoxic cells were maintained in an incubator infused with 2% O_2, 5% CO_2, and 93% nitrogen. To knock down LEENE or LEO1, cells were transfected with scrambled control LNA or siRNA or LNA targeting LEENE or LEO1 or MYC siRNA (Qiagen) using Lipofectamine-RNAiMAX transfection reagent (Thermo Fisher Scientific) in Opti-MEM (Thermo Fisher Scientific) according to manufacturer's protocol and as published (3). To overexpress LEENE, an adenoviral vector-driven expression of the predominant transcript of human LEENE (NR_026797.1) as previously described (3) was used to infect ECs or inject into *leene*-KO mice. Ad-GFP was used as a vector control.

Tube formation, scratch, and spheroid sprouting assays

The tube formation assay was performed as previously reported (4). Briefly, HMVECs were plated on a Matrigel (BD Pharmingen)-coated 24-well plate, incubated for 8 hours in 5% CO_2 at 37 °C, and examined for capillary tube formation under an inverted microscope and photographed. Three randomly selected views were captured, and the formed tubes were counted. For the scratch assay, HMVEC were seeded onto 24-well plates and grown to confluence. Cell monolayers were carefully wounded with a 200-µl pipette tip to generate a cut of ~1 mm in width. After two washing steps, cells were incubated for 24 hours and area lacking cells determined.

The 3D spheroid sprouting assay was performed as previous described (5). HMVECs cultured to confluency were trypsinized using 0.25% Trypsin/0.53 mM EDTA and counted. Enzyme activity was neutralized with culture media and centrifuged at 200 rcf for 5 minutes. The media was aspirated, and the cells were resuspended at 10^6 cells/mL in fresh media. To form homogeneous aggregates of 500 cell per aggregate, 125 µl of the resuspended cells were added to 3.875 mL of HMVEC media. One mL of 0.3% (W/V) methylcellulose (Sigma M0512-100G) in HMVEC media was then added to the suspension to bring the total volume to 5 mL, resulting in a final density of 500 cells per 20 µL. The cell suspension was then distributed onto the inside lid of a petri dish using a multichannel pipette to form rows of 20 µL droplets. The dish was then inverted, and 5 mL of PBS was added to the bottom of the dish. Cells were incubated overnight. On the next day, cell aggregates were collected, washed, and centrifuged at 100 rcf for 2.5 minutes. The aggregates were resuspended in pre-chilled Matrigel (Corning 356234) to allow for two aggregates per 20 µL. Aggregates were distributed in 20 µL droplets onto the bottom of the plate. The plate was then turned upside down to form hanging drops and placed into a larger petri dish (to maintain sterility), which was then placed in incubator for 30 minutes. After the Matrigel has begun to gel, the plate was removed from the larger dish and turned upright to allow another hour to fully set. The gelled

aggregates were then overlaid with HMVEC media containing 50 ng/mL of VEGF (Sigma V7259-10UG) and incubated for 3 days, with monitoring for sprouting.

Brightfield images of the sprouts were taken using an Amscope MU1000 camera and an Olympus IX50 microscope at 10X magnification. The images were then analyzed in FIJI (ImageJ) using the Sprout Morphology analysis tool (6). The images were first converted to 8-bit binary masks. Threshold values were then globally adjusted across all images to uniformly darken the background and highlight the aggregates and their sprouts. The images were then manually assessed for bubbles or other artifacts in the gel that the software could misconstrue as an aggregate. Pixel scale was determined using a hemocytometer, and this was applied globally to all images. Then the images were batch-run through the analysis package, where the software measured the aggregates and the sprouts.

Measurement of blood pressure and echocardiography

Blood pressure was measured using a noninvasive computerized tail-cuff system (Visitech, Apex, NC) as previously described (7). After the mice were placed in a plastic holder, the occlusion and sensor cuff were positioned on the base of the tail. All the mice were given at least 1 week to adapt to the system prior to blood pressure measurement. Blood pressure was measured at least 20 times in each mouse. Echocardiography was performed as previously described (8). Briefly, mice under conscious condition were used with a Vevo 3100 Ultrasound Imaging System (FUJIFILM VisualSonics). Multiple parameters including heart rate, left ventricular internal dimensions at end of diastole and systole (LVIDd and LVIDs), end-diastolic interventricular septal thickness (IVSd), and LV posterior wall thickness (LVPWd) were determined from the ventricular M-mode tracing.

Percentage fractional shortening (%FS) and ejection fraction (EF) were used as indicators of systolic cardiac function.

Histology, immunostaining, and immunoblotting

Histological examinations were mainly processed by the Solid Tumor Pathology Core at City of Hope. Skeletal muscle from mice was collected and fixed in 4% paraformaldehyde over-night. The fixed tissues were later dehydrated, sectioned into 4 μm paraffin slides, and subjected to Hematoxylin and Eosin (HE) staining. For immunofluorescent (IF) staining, antibodies against CD31 (Rat mAb #ab56299, Abcam, 1:200 dilution), Kdr (Rabbit mAb #2479, Cell Signaling Technology, 1:100 dilution), IB4 fluorescein (FL1201-.5. Vector laboratories, 1:100 dilute) and GFP (Rabbit mAb #2956, Cell Signaling Technology, 1:75 dilution) were used as primary antibodies. As secondary antibodies, Alexa Fluor555-conjugated goat anti-rat IgG (A-11007, Invitrogen, 1:200 dilution) or Alexa Fluor 488-conjugated goat anti-rabbit IgG (A-11037, Invitrogen, 1:200 dilution) were used as appropriate. Nuclei were stained with DAPI (P36935, Invitrogen). Images were taken using a ZEISS Axio Observer. For immunoblotting of LEO1, antibody against LEO1 (Rabbit polyclonal #A300-175A, BETHYL, 1:1000 dilution) was used as primary, and anti-rabbit (7074S, Cell Signaling Technology, 1:5000 dilution) was used as the secondary antibody.

RNA isolation, RT-qPCR analyses

RNA was extracted from cells and tissues using TRIzol (Thermo Fisher Scientific). The total RNA was reverse transcribed using PrimeScript RT Master Mix (Takara), and cDNAs were used for qPCR analyses using the primers listed in Supplemental Table 5. Samples were subjected to qPCR

using iTaq Universal SYBR Green Supermix on a CFX Connect system (BioRad). β-actin was used as the internal control in human and 36B4 in mouse samples.

Single-molecule RNA fluorescent in-situ hybridization (smFISH)

smFISH was performed on human mesenteric arteries using the RNAscope™ Multiplex Fluorescent V2 Assay (ACDBio). Cells grown and treated on coverslips were fixed with 4% paraformaldehyde (PFA) for 30 minutes at room temperature, ethanol dehydrated, pre-treated with hydrogen peroxide for 10 minutes at room temperature, and permeabilized with Protease III (1:10 dilution) for 30 minutes at room temperature prior to probe hybridization. RNAscope® Probe – Hs-linc00520 (502321, ACS) was used to detect human LEENE. Following probe hybridization, the RNAscope assay was developed following the recommended protocol.

Subcellular fractionation

HUVECs were collected in 200 µl cold cytoplasmic lysis buffer (0.15% NP-40, 10mM Tris pH 7.5, 150mM NaCl) and incubated on ice for 5 minutes. The lysate was layered onto 500 µl cold sucrose buffer (10mM Tris pH 7.5, 150mM NaCl, 24% sucrose weight by volume) and centrifuged. The supernatant containing cytoplasmic component was quickly added to TRIzol LS for RNA extraction. The nuclear pellet was gently suspended into 200 µl cold glycerol buffer (20mM Tris pH 7.9, 75mM NaCl, 0.5mM EDTA, 50% glycerol, 0.85mM DTT). Cold nuclei lysis buffer (20mM HEPES pH 7.6, 7.5mM MgCl2, 0.2mM EDTA, 0.3 M NaCl, 1M urea, 1% NP-40, 1 mM DTT) was added and the mixture vortexed and centrifuged. The supernatant containing the nucleo-plasmic fraction was mixed with TRIzol LS (Thermo Fisher Scientific) for RNA extraction. Cold PBS (50 µl) was added to the remaining pellet and gently pipetted. After vigorous vortexing to

resuspend the chromatin, chromatin-associated RNA was extracted by adding 100 μl chloroform and TRIzol reagent. RNA samples from three different fractions were dissolved with same amount of RNase-free water, and same volume of RNA was used for reverse-transcript and qPCR.

ChIRP-seq

ChIRP was performed as described (3, 9). HUVECs were fixed with 1% glutaraldehyde for 10 minutes at room temperature. The pelleted cells were lysed and sonicated for 10 minutes using a "30s ON, 30s OFF" program. The sonicated samples were then centrifuged and 1% of the supernatant was taken as input of ChIRP-DNA-seq and another 1% of the supernatant was taken as the input. About 100 pmol of the probes were hybridized with the residual supernatant at 37 °C for 4 hours, followed by incubation with streptavidin-conjugated magnetic beads for another 30 minutes. Following several rounds of washing, DNA was isolated from the ChIRP precipitates and subsequently used for sequencing. Subsequently, ChIRP-seq libraries were constructed using the KAPA HyperPrep Kit (Roche Diagnostics) following the manufacturer's manual. The DNA was quantified using Qubit double-stranded DNA High sensitivity assay (Thermo Fisher Scientific).

Liquid chromatography/Mass spectrometry

After ChIRP, proteins were reduced with 10 mM tris(2-carboxyethyl) phosphine, alkylated with 30 mM iodoacetamide, and digested with 1 ug Trypsin/LysC (Promega) overnight at 37 °C. Peptides were purified with Oasis HLB columns. Mass spectrometry was performed on an orbitrap Fusion Tribrid instrument (Thermo) equipped with an Easy-nLC 1000 HPLC system, a 75 μm by 2 cm PepMap C18 trapping column, a 75 μm by 50 cm PepMap RSLC C18 analytical column, and an Easy-Spray ion source (Thermo). Peptides were separated by a 1 h gradient from 0.1%

formic acid, 3% acetonitrile to 0.1% formic acid, 30% acetonitrile. Precursor ion scans were acquired in the orbitrap and CID fragments were acquired in the linear ion trap in rapid mode. Data analysis was performed using Proteome Discoverer with the Sequest search engine (Thermo) and Scaffold (Proteome Software).

ChIP assay

ChIP assays were performed as previously described (10). Briefly, HUVECs were treated with 0.75% formaldehyde for 20 minutes at room temperature. Fixation was stopped by adding 125 mM glycine and the cells were collected. The pelleted cells were lysed and sonicated for 4 minutes using a "30s ON, 30s OFF" program at 4 °C. The sonicated samples were then centrifuged and 1% of the supernatant was taken as input. After sonication, the chromatin was incubated with rabbit anti-human H3K4me3 (Rabbit polyclonal #39159, Active Motif) or anti-human H3K27ac (Rabbit polyclonal #39133, Active Motif) conjugated to prewashed Protein A Dynabeads (Thermo Fisher Scientific). Protein and RNA were digested by proteinase K and RNase A, respectively. The purified chromatin DNA was then used as the template for qPCR.

RNA IP (RIP) and Co-IP

RIP was performed as previously described (3). Briefly, cells were UV-crosslinked (400 mJ/cm^2) and collected with cold PBS, then lysed with a buffer containing 50 mM Tris-HCl, pH 7.4, 100 mM NaCl, 1% NP-40, 0.1% SDS, 0.5% sodium deoxycholate ice for 15 mins with protease inhibitor cocktail (Roche). Protein A Dynabeads (10008D, Invitrogen) incubated with IP antibodies against LEO1 (Rabbit polyclonal #A300-175A, BETHYL) or MYC (Rabbit mAb #18583S, Cell Signaling Technology) or IgG (Normal Rabbit #2729S, Cell Signaling Technology)

at room temperature for 2 hours, then incubated with cell lysis 4°C overnight, followed by washed with cold high salt buffer. RNA in the immunoprecipitates were extracted with Trizol. For CO-IP, protein A beads were washed with RIPA lysis buffer for 30min at 4°C, the cells were lysed with RIPA lysis buffer and the supernatant was added into the beads buffer and incubated with MYC antibody (Rabbit mAb #18583S, Cell Signaling Technology), LEO1 antibody (Rabbit polyclonal #A300-175A, BETHYL), or IgG (Goat HRP-linked antibody #7074S, Cell Signaling Technology) overnight at 4°C with gentle shaking. The immunoprecipitated proteins were resolved by Western blotting.

Nascent RNA pulldown

To capture nascent RNA, newly synthesized mRNA was isolated using the Click-iT Nascent RNA Capture Kit (Thermo Fisher Scientific). HUVECs were synchronized with 2% FBS in M199 medium for 8 hours, followed by incubation in 0.2mM of 5-ethymyluridine (EU, an alkyne-modified uridine analog which is incorporated into the nascent RNA) for another 24 hours, and total RNA was isolated using TRIzol. A copper-catalyzed click reaction was performed using 5 µg RNA with 0.5mM azide-modified biotin. The mixture was incubated at room temperature for 30 minutes following RNA precipitation. Biotin-labeled EU-RNA was then pulled down by mixing with Streptavidin T1 magnetic beads at room temperature for 30 minutes and the unbound RNA was washed away. The cDNA synthesis was performed directly on the beads using the Superscript VILO cDNA synthesis kit (Thermo Fisher Scientific), followed by qPCR analysis.

Supplemental Figures and Legends

Supplemental Figure 1. Heatmap of full list lncRNA expression in ECs treated by multiple stimuli as indicated, ranked by fold-change (FC) as shown in Figure 1A.

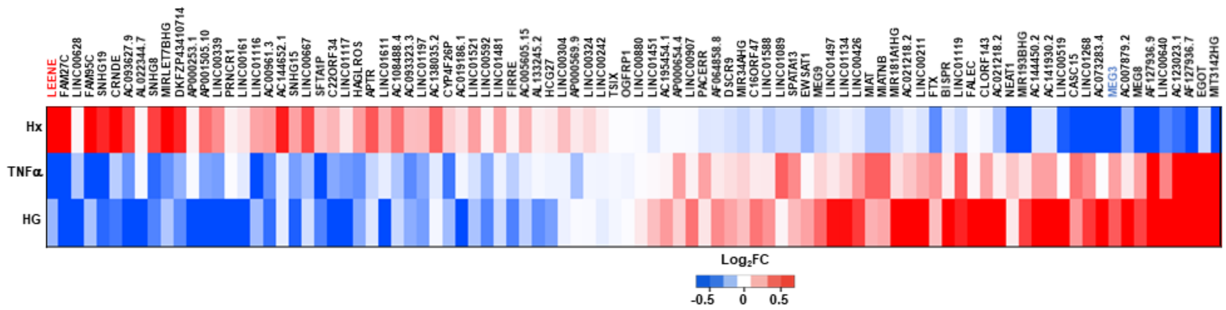

Supplemental Figure 2. Enriched pathways in ECs with LEENE knockdown (KD). LEENE was knocked down in HUVECs using LNA GapmeRs and transcriptome was profiled using RNA-seq as in Figure 2. The top 30 enriched biological pathways in all DEGs in ECs with LEENE KD.

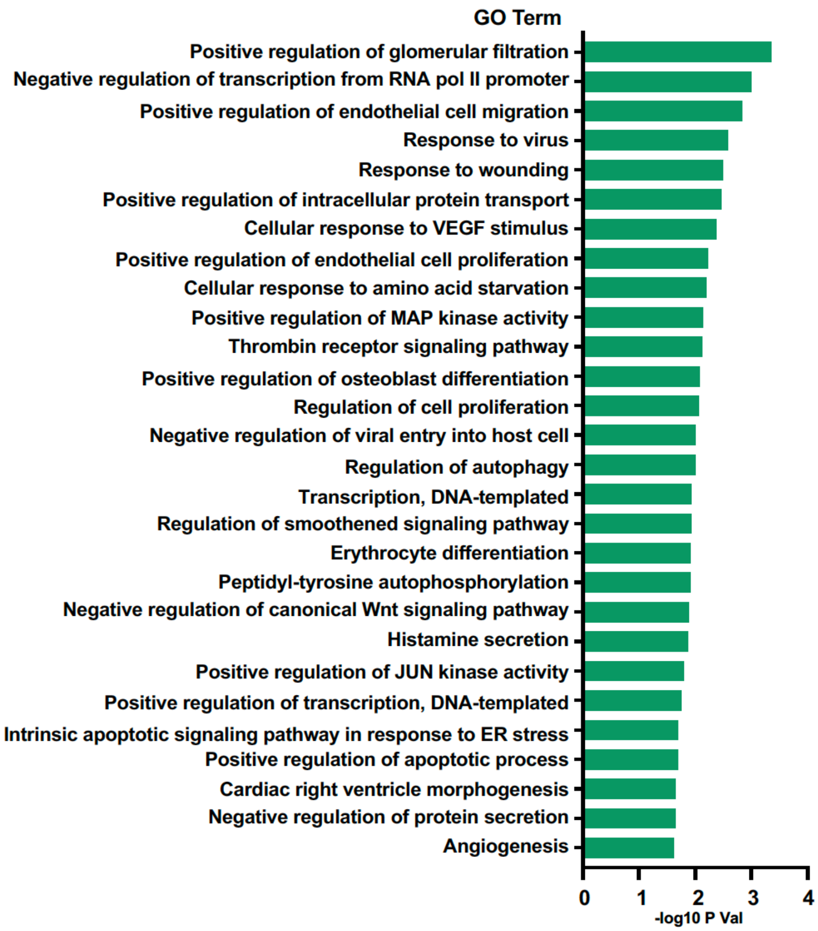

Supplemental Figure 3. Enriched pathways in ECs with LEENE KD, separated by down or upregulated DEGs. DEGs identified as in Figure 2 were separated by down or upregulation and then subjected to pathway enrichment analysis. The top 30 enriched biological pathways in the downregulated (A) and upregulated (B) DEGs in ECs with LEENE knock down are shown.

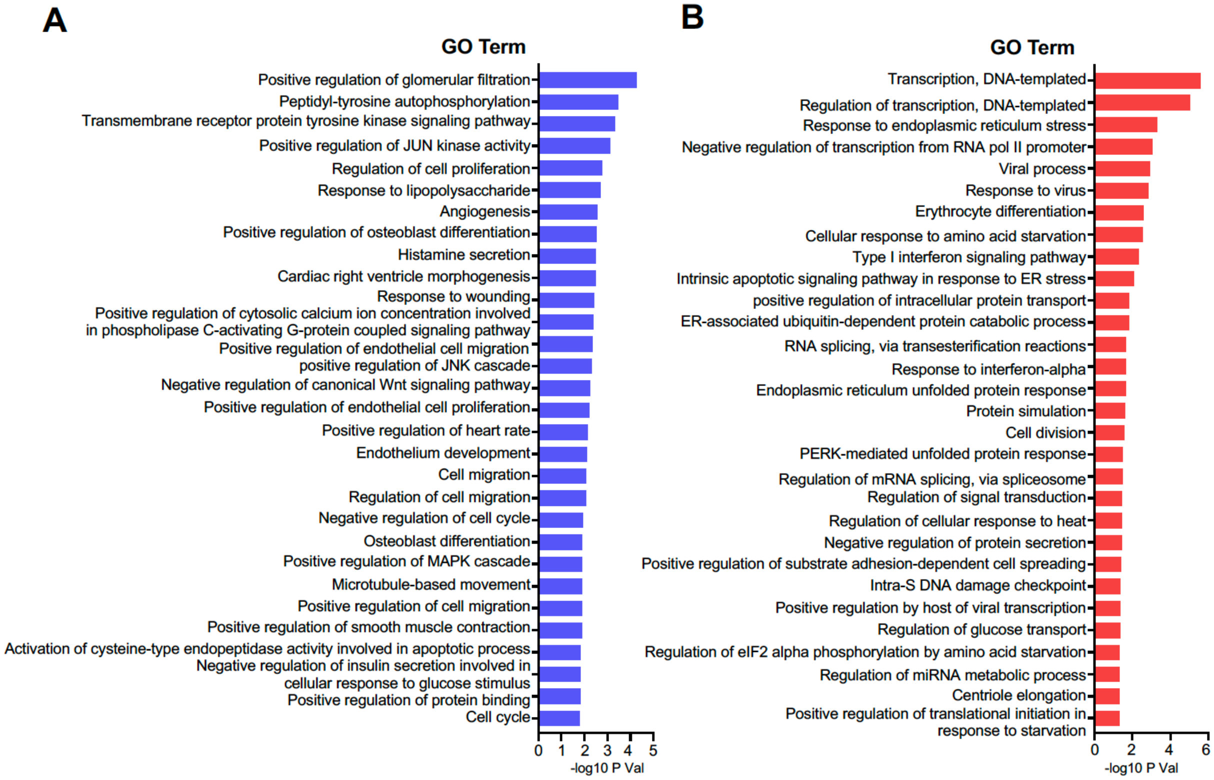

Supplemental Figure 4. Targeting strategy and cutting efficiency of sgRNAs used to create *leene*-KO mice. (A) Knockout strategy in mouse genome: the deleted region in mouse relative to human LINC00520 is shown. Bottom tracks are from HUVEC ChIP- and RNA-seq data available on ENCODE. (B, C) Small guide RNAs (SgRNAs) were designed to cleave the 5' (in B) and 3' (in C) of the mouse syntenic region of *LEENE.* Cutting efficiency of 6 candidate sgRNAs were assessed by surveyor assay, as resolved on 1.5% agarose gel. Sg3 and Sg5 were used to create the *leene*-KO mice.

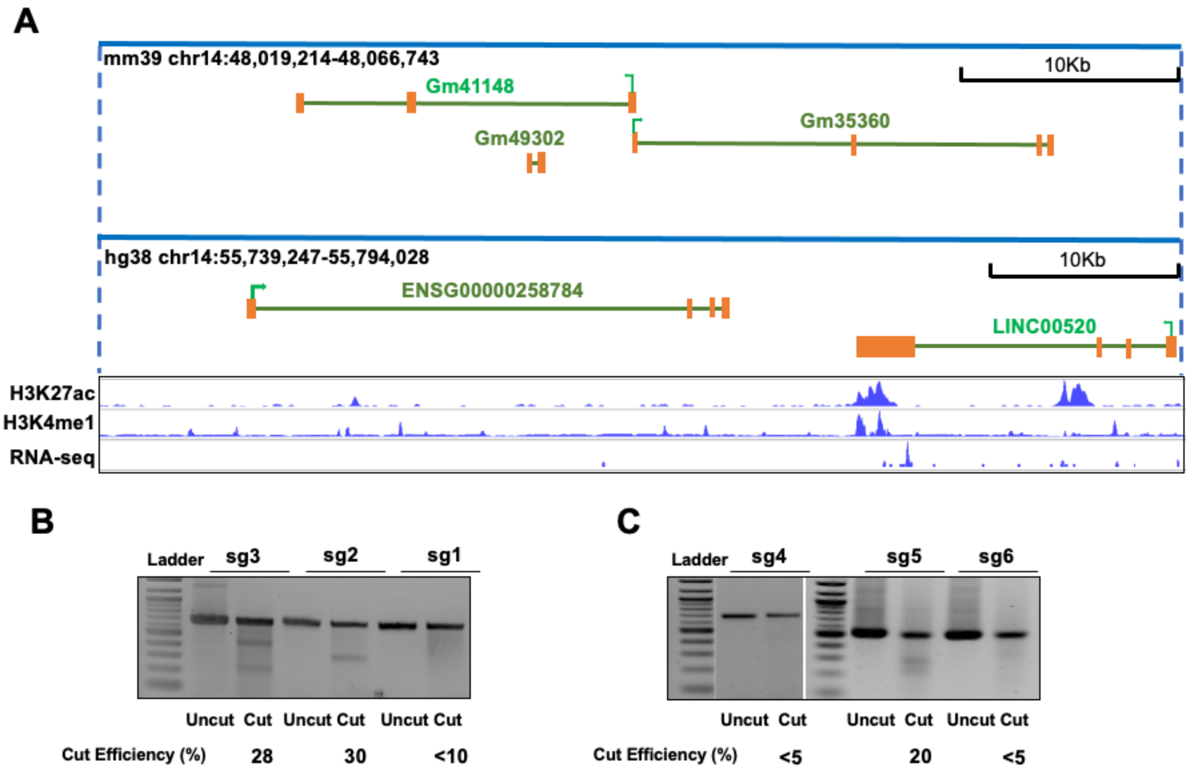

Supplemental Figure 5. Body composition, GTT, and diastolic BP in male *leene*-KO mice. Male *leene*-KO and their WT littermates were fed chow diet for 24 weeks or HFHS diet for 16 weeks starting at 8 week-old and subject to measurements of (A) body composition, (B) ejection fraction (EF) and (C) diastolic BP (n=3-11/group). Data are represented as mean ± SEM. *P=0.04, 0.04, 0.05 and 0.05 (from left to right) as compared between indicated groups based on two-way ANOVA followed by Tukey's test.

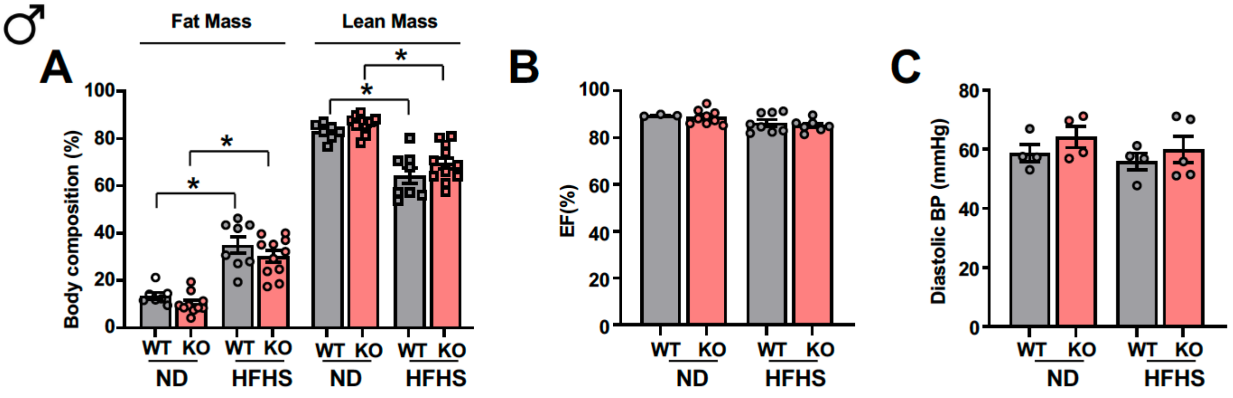

Supplemental Figure 6. Metabolic and cardiovascular phenotyping of female *leene*-KO mice. Female mice fed chow or HFHS diet for 16 weeks starting from 8 week-old were used for measurements of (A) body weight, (B) body composition (C) glucose tolerance, (D,E) cardiac function, and (F,G) BP (n=3-10/group). Data are represented as mean ± SEM. *P=0.04 and 0.05 (left to right) based on two-way ANOVA followed by Tukey's test in (B).

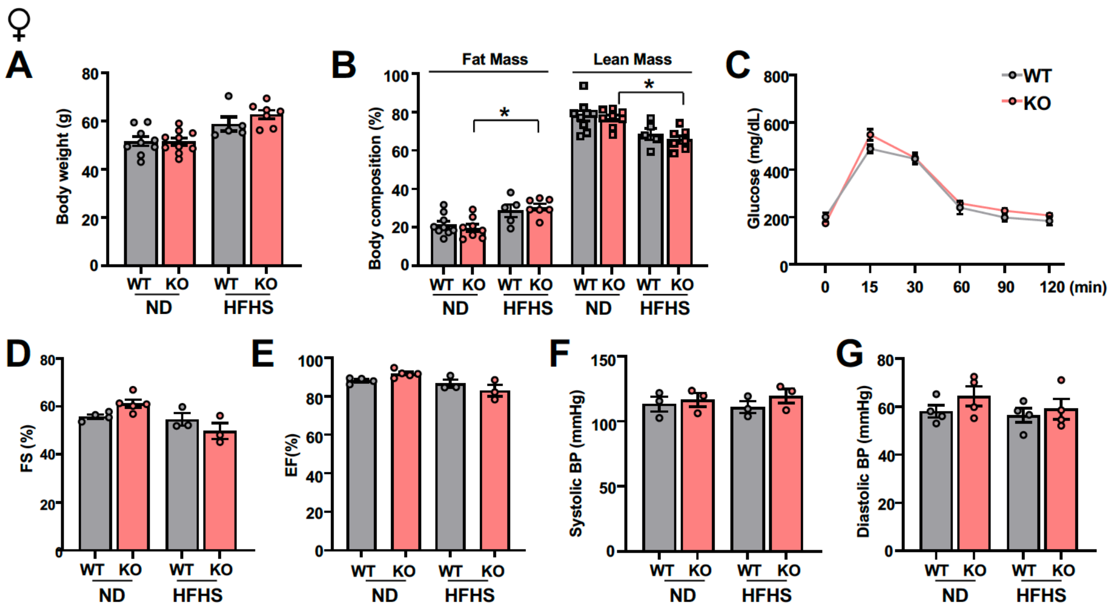

Supplemental Figure 7. Comparable flow recovery in WT and *leene*-KO mice under chow diet. Chow-fed 24-week-old male (A and B) and female (C and D) mice were subjected HLI. Flow perfusion was measured right after the surgery (D0) and weekly for 4 weeks. Representative flowgraphy images (A and C) and quantitative analysis of the perfusion ratio (ischemic vs non-ischemic limb) (B and D). Male: WT=8 and KO=10 mice/group; female: WT=4 and KO=7 mice /group. Data are represented as mean ± SEM.

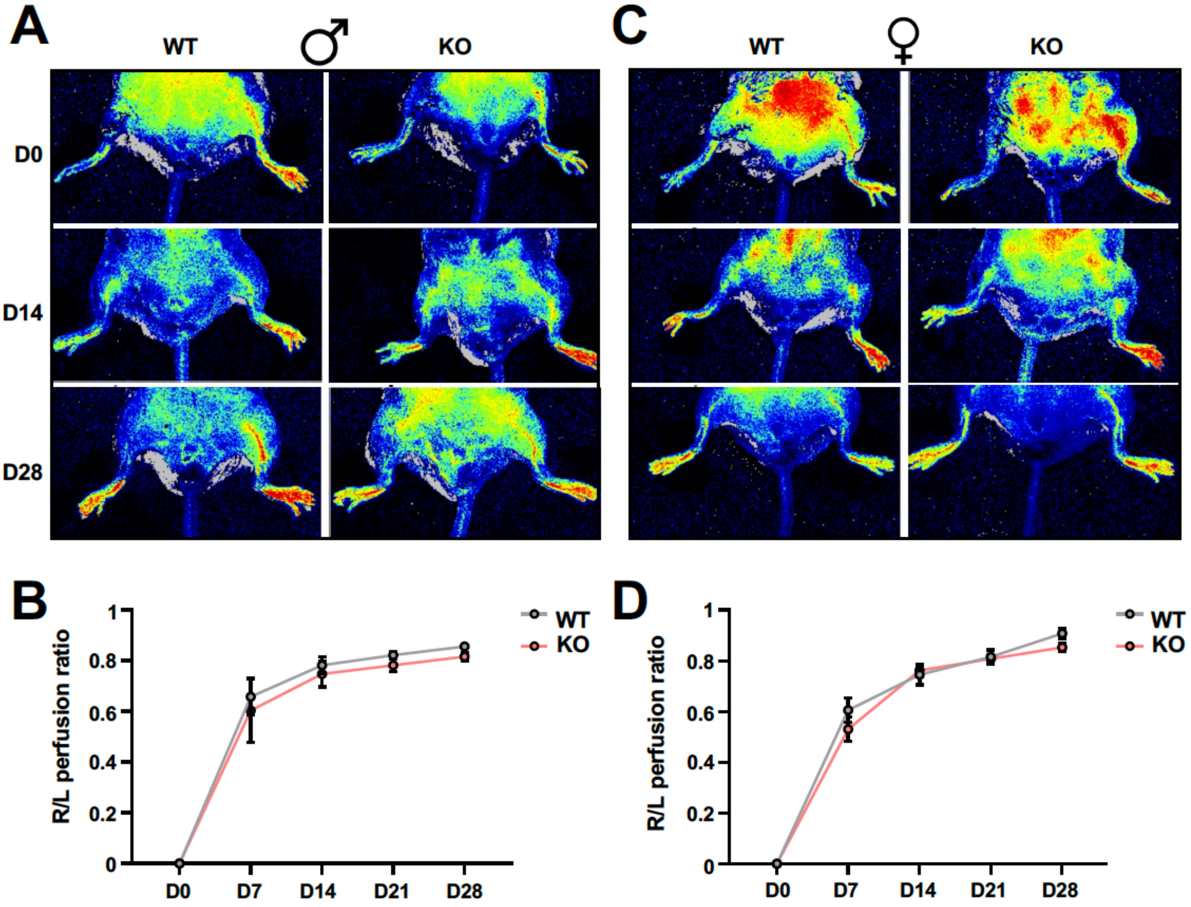

Supplemental Figure 8. smRNA FISH of LEENE and IB4 staining. smRNA FISH of LEENE IB4 staining of capillaries of ischemic hindlimb muscle from KO+Ad-LEENE mice, with DAPI counterstain of nucleus. KO+GFP mice serve as a control. Arrows indicate colocalization of LEENE and IB4 signals. Scale bar = 50 µm. The merge image of KO-LEENE is also shown in Figure 5C.

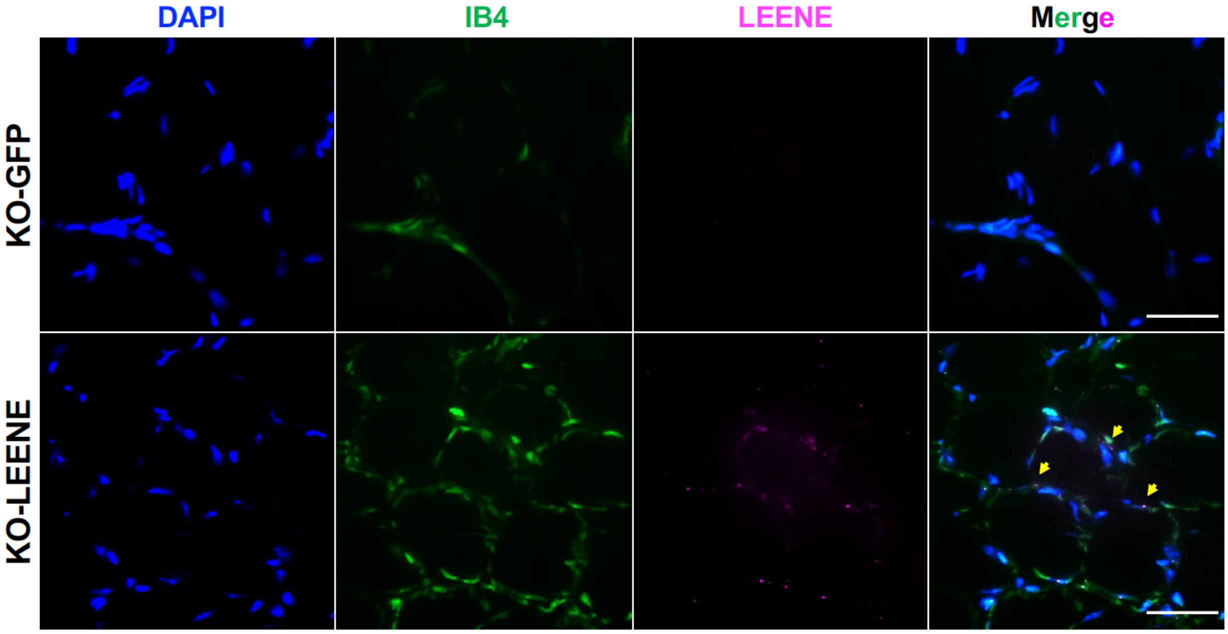

Supplemental Figure 9. Rescue effect of Ad-LEENE injection in *leene*-KO mice subjected to HLI. Mice were fed HFHS diet starting from 8-week-old for 16 weeks and then subjected to HLI, followed by intramuscular injection of Ad-GFP or Ad-LEENE. Representative flowgraphy images showing hindlimb recovery of male (A) and female (C) mice on day 0, 4, and 7 post HLI. (B) Quantification of capillary density stained by IB4 from three groups (n=3-5/group). (D) Quantitative analysis of hindlimb flow perfusion in the female mice (n= 4 mice/group). Data are represented as mean ± SEM. *P=0.002, P=0.01 in (B) and *P =0.0001 between indicated groups in (D) based on ANOVA followed by Tukey's test. The flowgraphy images of day 7 post HLI are also shown in Figure 5E.

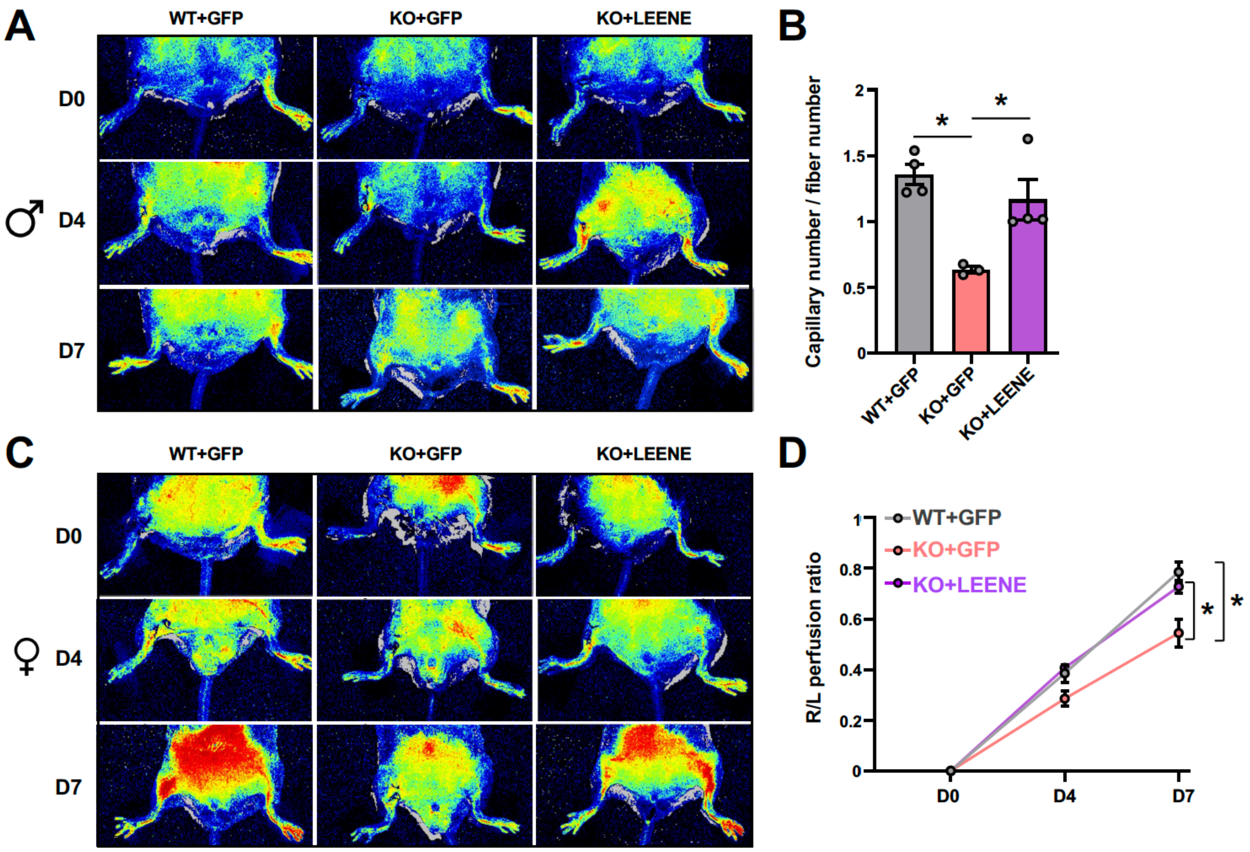

Supplemental Figure 10. Enriched pathways with LEENE rescued genes. (A) Top 6 Biological Pathway terms from pathway enrichment analysis with GO term among LEENE-rescued genes, namely the overlap between downregulated by *leene* KO (WT+GFP vs KO+GFP) and upregulated by LEENE overexpression in KO (KO+GFP vs KO+LEENE), plotted with P-value and gene count. (B) WT and KO mice were subjected to HLI fed a HFHS diet and received Ad-GFP or Ad-LEENE as in Figure 5. qPCR of *KDR* mRNA in ischemic tissues. n=3/group. Bar graphs represent mean ± SEM. *P =0.003 and 0.05 between indicated groups based on ANOVA followed by Tukey's test.

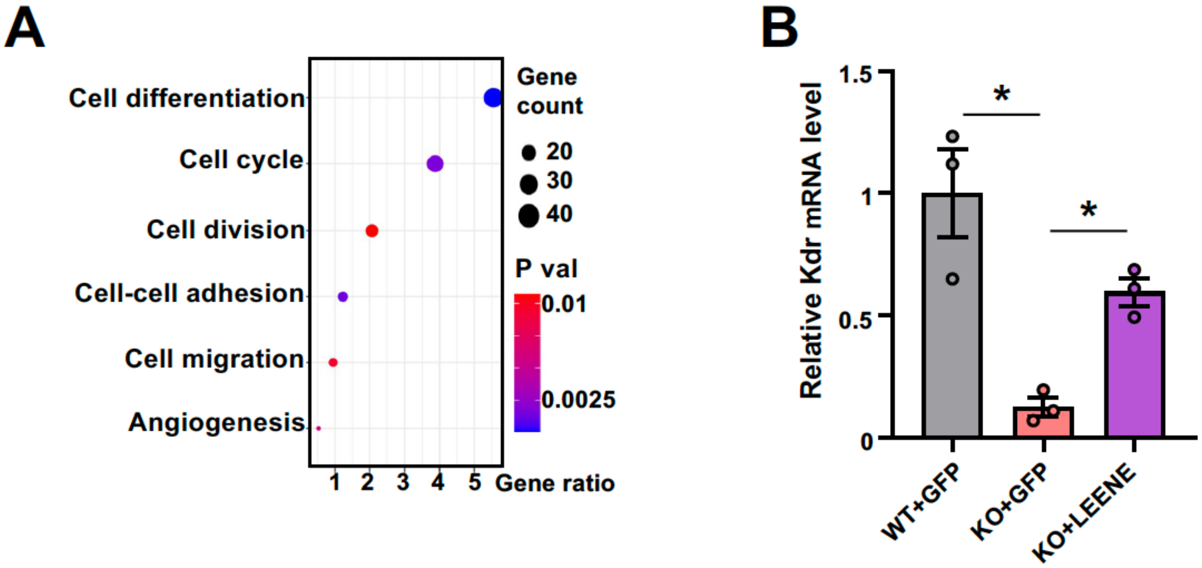

Supplemental Figure 11. Enriched pathways in VSMCs from ischemic muscles with LEENE OE. *Leene*-KO mice were subjected to HLI and Ad-GFP/LEENE injection. The hindlimb muscles underwent scRNA-seq analysis as presented in Figure 7. DEGs in VSMCs identified from scRNA-seq were subjected to pathway enrichment analysis. The top 30 enriched biological pathways are shown.

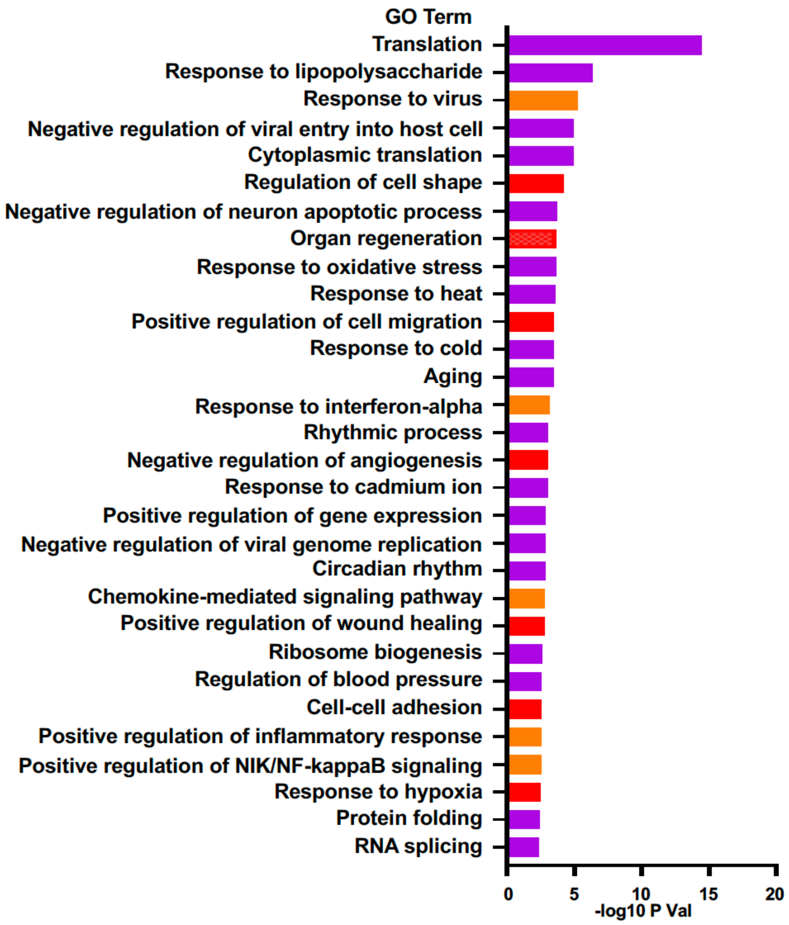

Supplemental Figure 12. Ligand-receptor interactions between ECs and the other cell types in hindlimb by LEENE overexpression. Network visualization of ligand-receptor connectivity between ECs and VSMC, macrophage (Mϕ), or fibroblasts (Fibro), based on the expression of ligands (blue) and receptors (red) in the scRNA-seq data as in Figure 7, with gene names showing for all involved ligands and receptors.

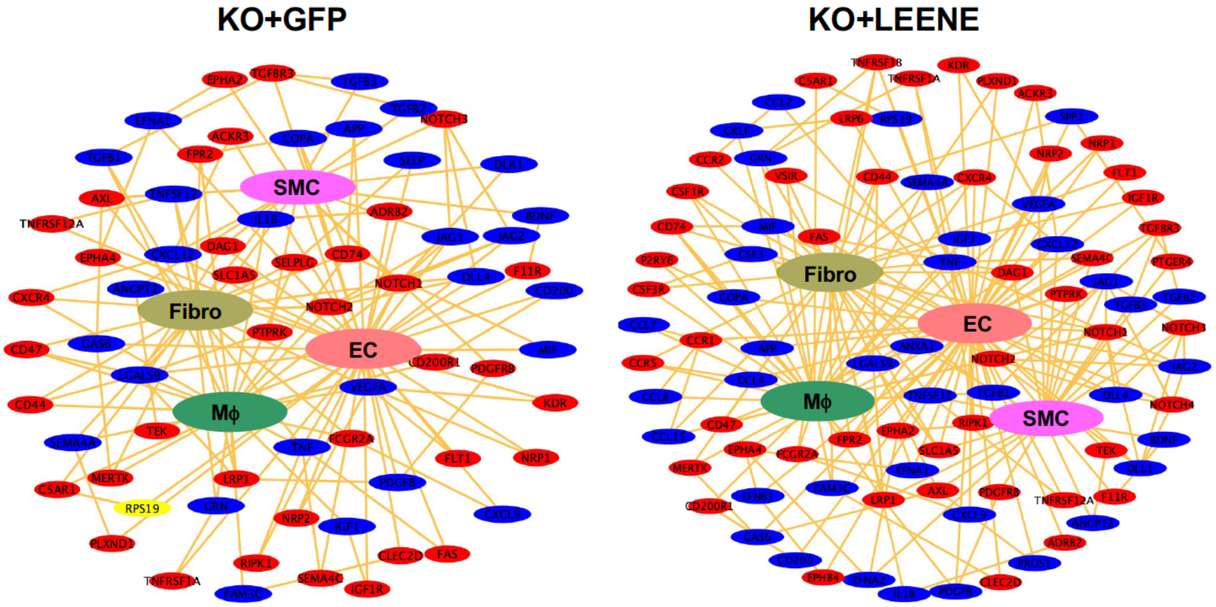

Supplemental Figure 13. qPCR of LEENE in different subcellular compartments. HUVECs were infected with Ad-GFP or Ad-LEENE for 72 hours, followed by subcellular fractionation to obtain cytoplasm (Cyt), nucleus (Nuc), and chromatin-bound fractions (Chr) and qPCR (n=3/group). Data are represented as mean ± SEM. *P =0.05, 0.02, 0.0001 based on t test.

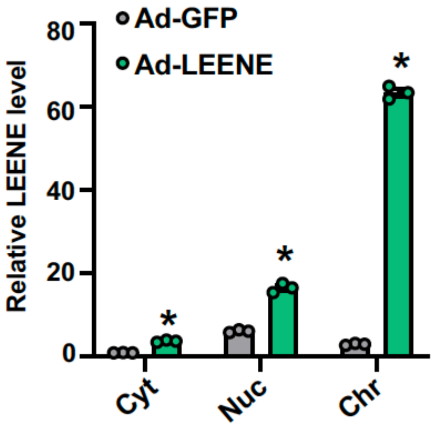

Supplemental Figure 14. ChIRP-qPCR of LEENE-bound DNA. qPCR was performed with chromatin pulldown using probes specific for LEENE or LacZ RNAs. eNOS and KDR promoters were detected in the DNA extracted from precipitates (n=2/group). Data are represented as mean ± SEM.

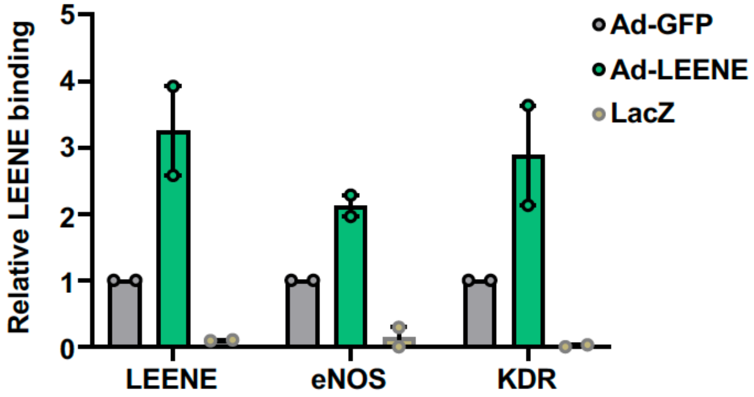

Supplemental Figure 15. Pie plot showing the down and up-regulated genes due to LEENE-KD (revealed by RNA-seq) in relation to LEENE binding (based on ChIRP-seq).

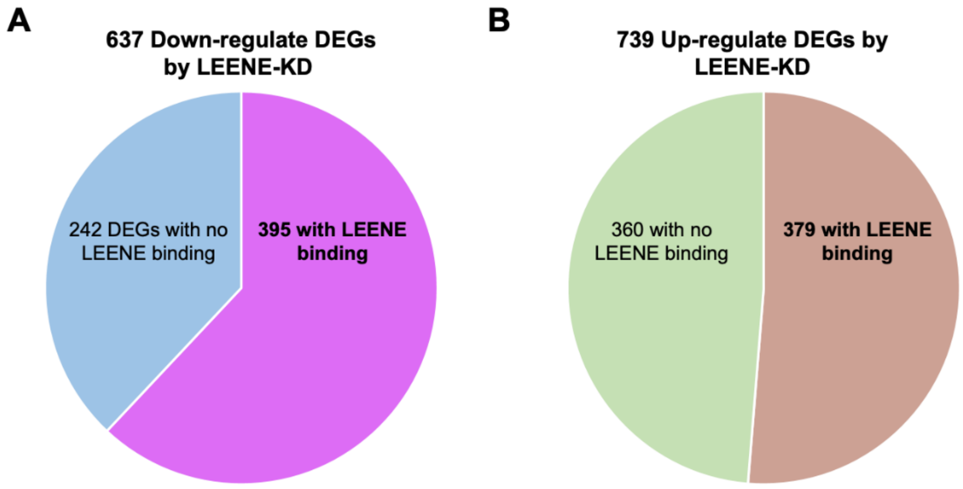

Supplemental Figure 16. Bioinformatic workflow to identify putative genes that are positively regulated by LEENE through interaction.

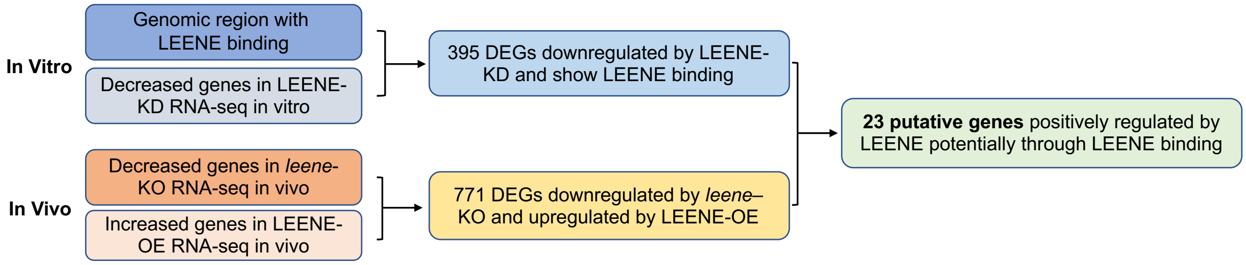

Supplemental Figure 17. ChIRP-mass spectrometry (MS) identified candidate LEENE-interacting proteins.

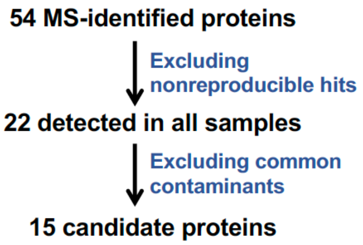

Protein	Full Name	Accession Number	Molecular Weight (KD)
LEO1	RNA polymerase-associated protein LEO1 isoform 1	NP_001273359.1	75
ZNF804A	Zinc finger protein 804A	NP_919226.1	137
TTN	Titin isoform IC	NP_001254479.2|	3994
ANXA1	Annexin A2 isoform 1	NP_001002858.1	40
EEF1A1	Elongation factor 1-alpha 1	NP_001393.1	50
KIF20B	Kinesin-like protein KIF20B isoform 2	NP_057279.2	206
GAPDH-1	Glyceraldehyde-3-phosphate dehydrogenase isoform 1	NP_002037.2	36
GZMB	Serine protease 55 isoform 2 precursor	NP_001183949.1	31
PLCB1	1-phosphatidylinositol 4,5-bisphosphate phosphodiesterase beta-1 isoform	NP_001124432.1	189
SNCA	Alpha-synuclein isoform NACP140	NP_000336.1	14
CCDC66	Coiled-coil domain-containing protein 66 isoform 2	NP_001012524.4	106
VIM	Vimentin isoform X1	NP_003371.2	54
ACTB	Actin, cytoplasmic 1	NP_001092.1	42
TUBA1A	Tubulin alpha-1A chain isoform 2	NP_001257329.1	50
TUBB	Tubulin beta chain isoform b	NP_821133.1	50

Supplemental Figure 18. LEENE interacts with LEO1. (A) Validation of LEO1 antibody for IP. HUVEC total protein lysates were used for LEO1 IP and LEO1 protein was detected using immunoblotting in the IP beads and flow-through. (B) ChIRP was performed with ECs infected by Ad-GFP/Ad-LEENE in biological replicates. All 10 probes were used. LEO1 was detected using western blotting. (C) Odd and even probes were used in ChIRP followed by LEO1 detection using immunoblotting. In another ChIRP sample with all 10 probes, RNase was added to degrade RNA.

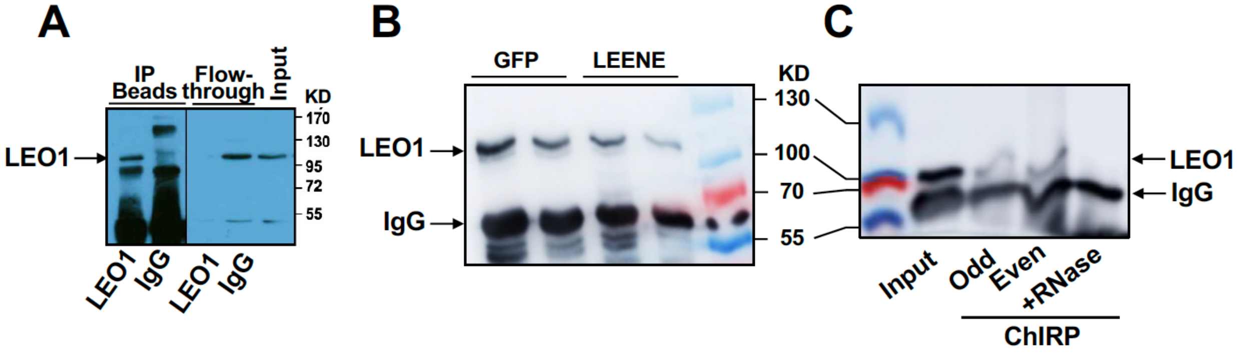

Supplemental Figure 19. Genes consistently downregulated by LEENE-KD in cultured ECs and by *leene*-KO in the EC-enriched fractions isolated from murine hindlimb muscles.

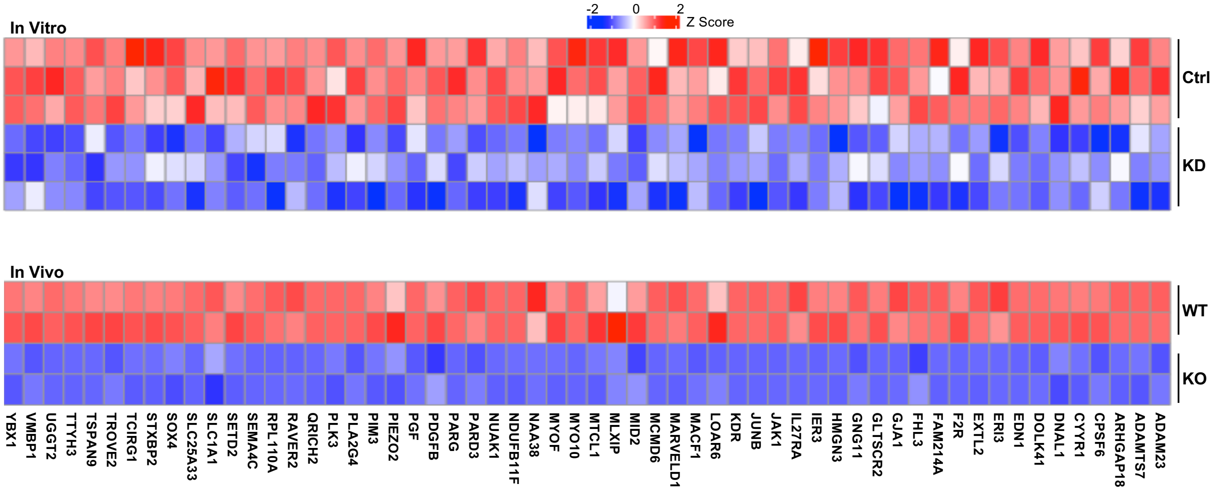

Supplemental Figure 20. Homology analysis using MEGA11 (A) and sequence alignment of human and mouse LEENE/leene (B).

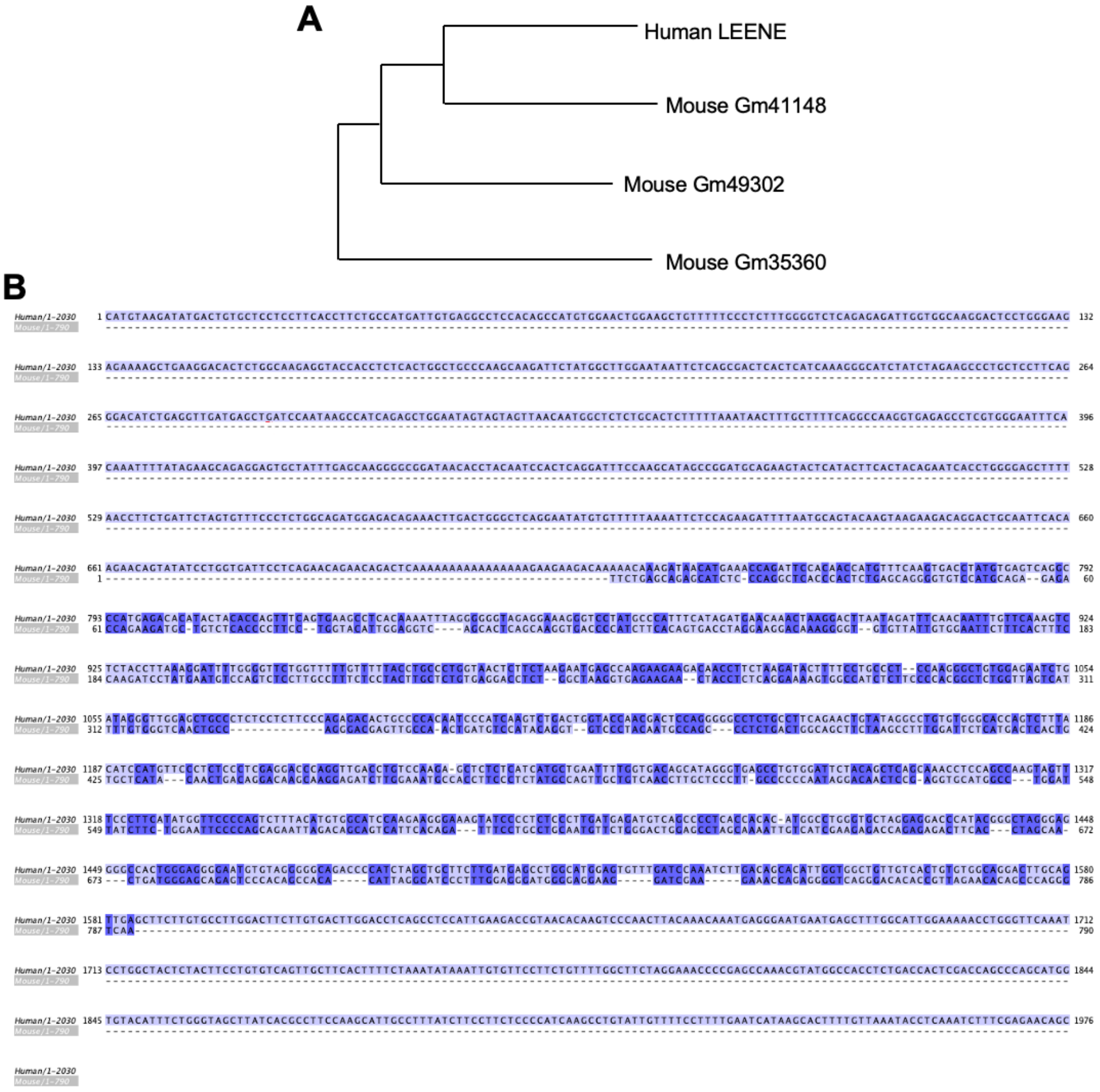

Supplemental Figure 21. qPCR of leene and two other transcripts encoded in the deleted locus in mouse tissues. n=3-5/group. Data are represented as mean ± SEM. *P< 0.0001 compared to expression of leene based on one-way ANOVA followed by Dunnett's test.

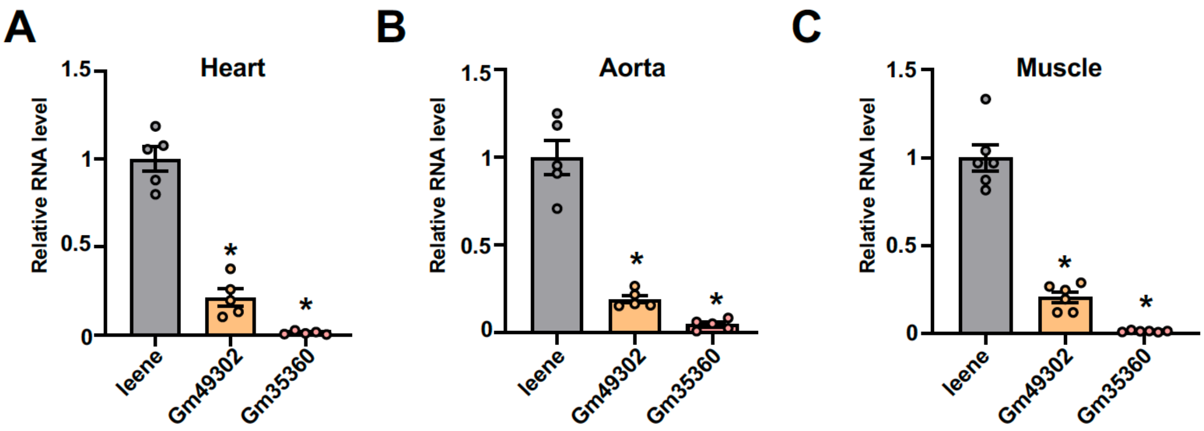

Supplemental Figure 22. LEENE activity in various cell types. H3K27ac ChIP-seq and RNA seq data on Encode showing LEENE locus and LEENE RNA transcription in multiple cell types.

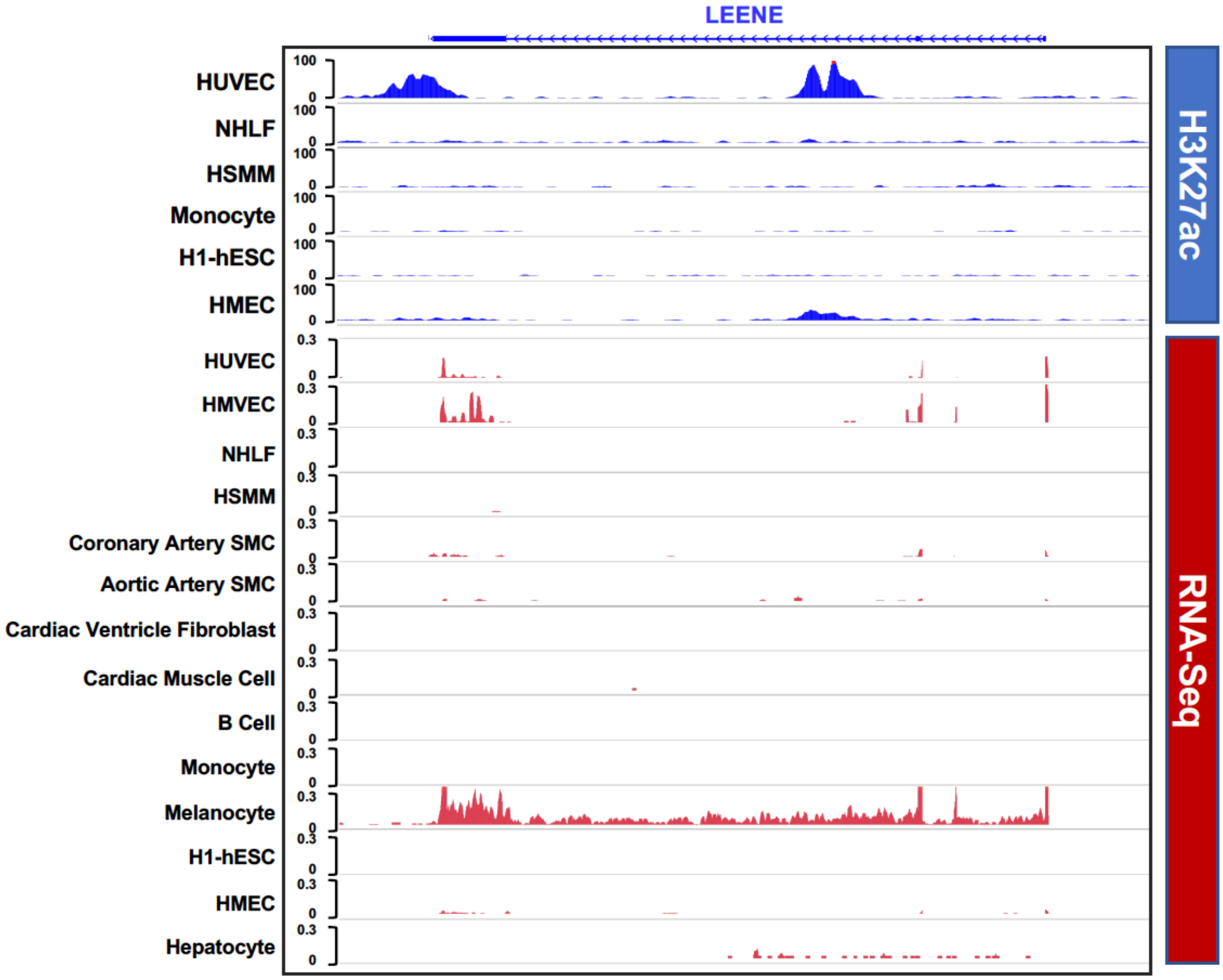

Supplemental Figure 23. Lack of effect of LEENE/leene gain- or loss-of-function in neighboring gene expression. qPCR of two neighboring protein coding genes in HUVECs transfected with LEENE LNA or infected by Ad-LEENE compared to respective controls (A,B) and in different tissues from WT and KO mice (C-E) and. n=3 in (A,B) and n=3-5/group in (C-E).

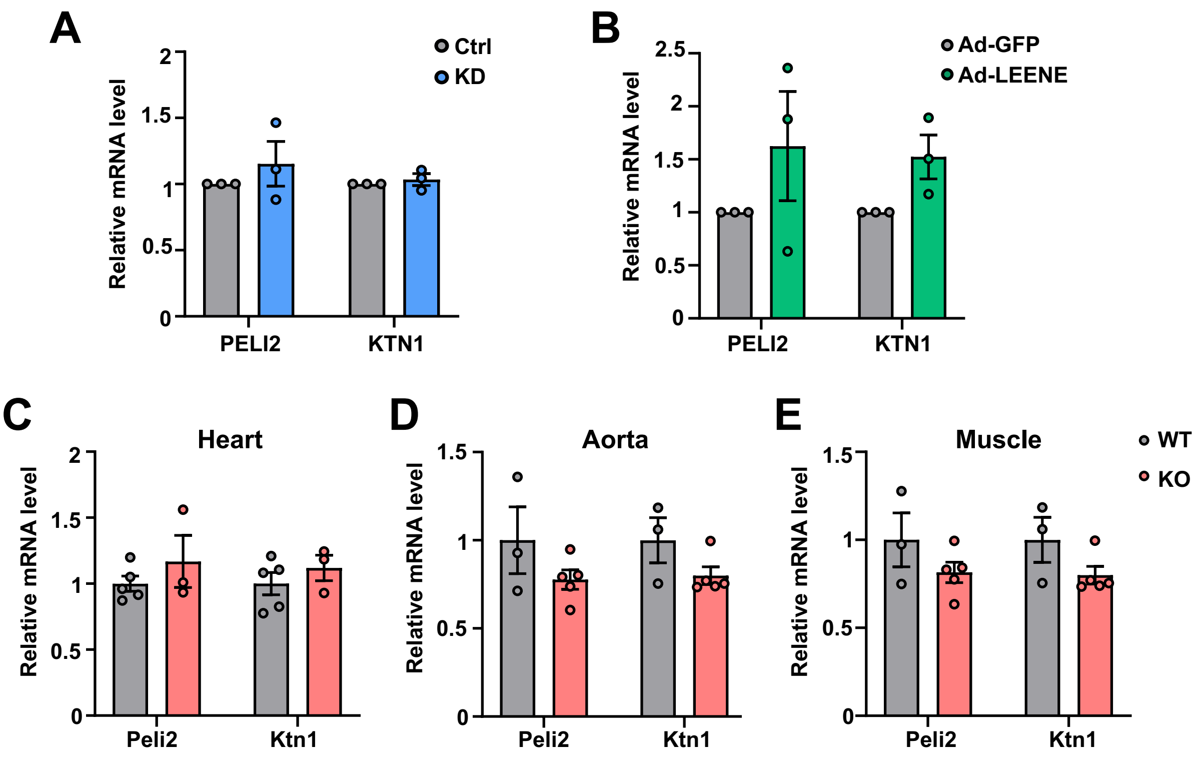

Supplemental Figure 24. LEENE promotes transcription of KDR and PGF. HMVECs were infected with Ad-GFP or -LEENE. (A) Nascent RNA was labeled by 5-ethynyluridine (EU), which was conjugated to biotin by a copper catalyzed reaction and subsequently pulled down by streptavidin beads. cDNA was synthesized on the beads, followed by qPCR. (B) ChIP was performed with H3K4me3 antibody, followed by qPCR for detection of DNA corresponding to KDR and PGF promoters. Data are represented as mean ± SEM. *P =0.05 as compared to Ad-GFP in all comparisons based on two-tailed Student's t test.

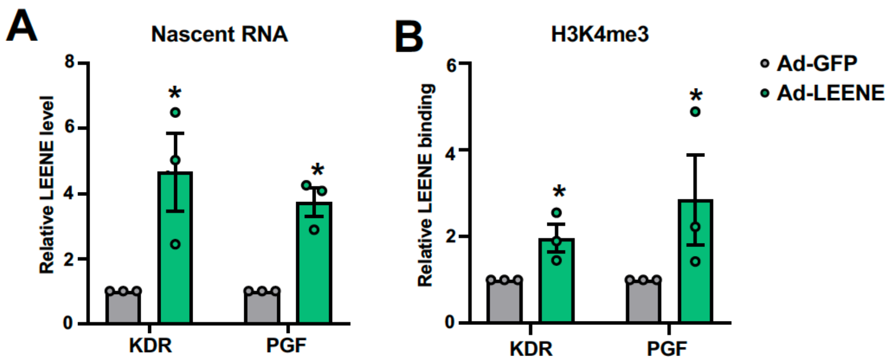

Supplemental Figure 25. Potential TFs involved in the LEENE-regulated transcriptome network. (A) IPA analysis showing the upstream network involving KLF2 and HIF1α. Turquoise arrows indicate transcriptional targets of KLF2 and purple arrows indicate targets of HIF1A. Green arrows indicate transcriptional regulation by both KLF2 and HIF1A. (B) Binding motifs for KLF4 and HIF1α enriched in LEENE-interacting DNA based on ChIRP-seq and TRANFAC analyses. (C) TF binding site prediction in *LEENE* locus. The yellow box indicates the putative promoter of *LEENE*. Vertical lines indicate putative TF binding sites for KLF (blue) and HIF (red).

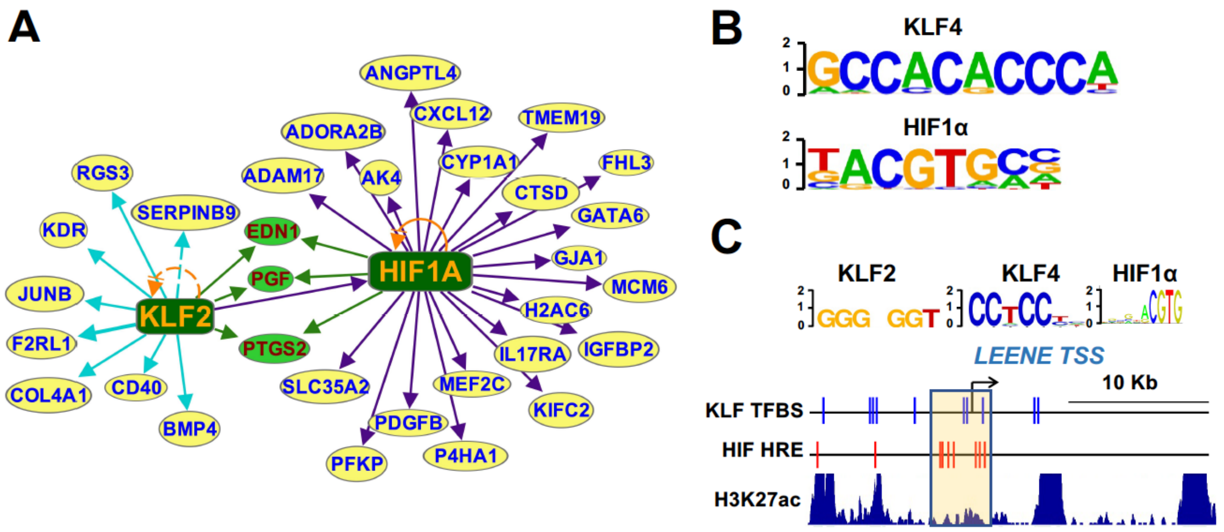

Supplemental Figure 26. KLF2 KD abrogated the effect of LEENE OE in inducing eNOS, KDR, and PGF. HUVECs were transfected with KLF2 or scramble siRNA and then infected with Ad-GFP and Ad-LEENE. *P=0.01, 0.003, 0.002, 0.0007, 0.0004, 0.01, 0.01, 0.02 (left to right) based on ANOVA followed by Tukey's test. Data are represented as mean ± SEM.

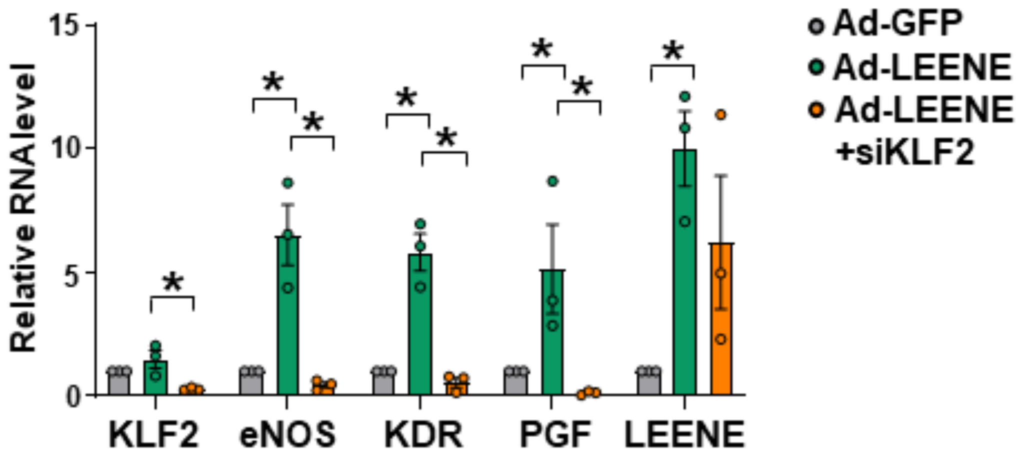

Supplemental Figure 27. Impaired retinal angiogenesis in WT vs *leene*-KO mice. (A) Representative images of αSMA (red) and IB4 (green) staining in the retina collected on postnatal Day 7. (B) Quantification of vascular area between WT and KO mice. (n=7-10/group). Data are represented as mean ± SEM. *P=0.015 based on t-test. Scale bar = 1 mm.

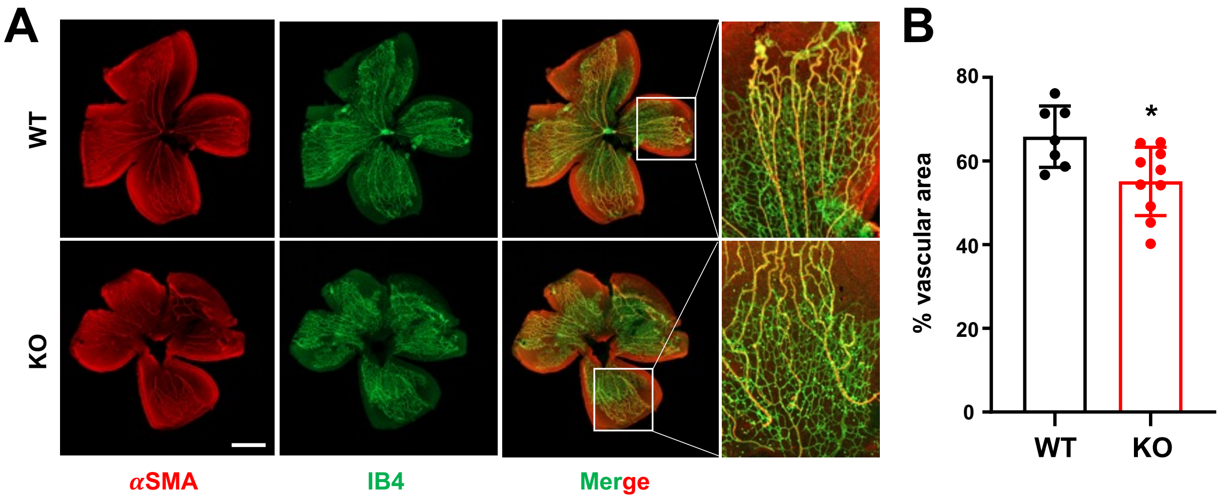

Supplemental Table 1. LncRNAs levels in multiple angiogenesis-related RNA-seq datasets

lncRNAs	NG (FPKM)	HG (FPKM)	Ctrl (TPM)	TNFα (TPM)	Nx (TPM)	Hx (TPM)
LINC00520	616.3	514.7	9588.9	8060.7	4872.5	9863.2
FAM27C	16.6	9.1	6351.7	4649.3	3528.4	6743.9
LINC00628	8.8	0.9	497.3	42.2	95.831	143.0
FAM95C	6.5	5.5	7153.3	5650.8	1575	3976.3
SNHG19	181	123.2	13324	10875	11068	12319.7
CRNDE	8.2	5.9	7950.5	7673.7	6260.1	8314.4
AC093627.9	10.2	5.4	2592.4	1735.2	2238.7	3371.2
AL022344.7	4.5	0.9	33.2	0	237.5	293.9
SNHG8	574.7	421.6	16679	15619	12951	14027.9
MIRLET7BHG	182.3	159.3	13726	12968	9407.5	10912.8
DKFZP434I0714	23.9	18.0	2379.7	1765.3	2235.1	3568.0
AP000253.1	1.68	0.4	419.5	403.0	254.65	342.3
AP001505.10	45.9	29.0	4914	4145.5	3281	4145.7
LINC00339	74.3	42.8	10093	9408.9	7434.3	8071.4
PRNCR1	4.0	1.3	28.4	28.697	0	87.0
LINC00161	1.1	0.4	504.3	353.8	0	161.0
LINC01116	102.8	87.3	4305.4	2547.3	7747.8	8241.6
AC009961.3	9.3	6.4	3227.1	2296.1	3688.5	4263.9
AC144652.1	8.4	7.8	976.1	836.9	1117.7	2504.1
SNHG15	193.9	128.6	9391.7	8693.7	8467.4	8964.3
LINC00667	477.9	444.7	8336.7	7892.9	6630.5	7727.0
SFTA1P	17.4	12.9	2118.6	608.8	1889.8	2231.0
C22orf34	45.1	27.7	2242.6	1516.8	4973.1	5375.6
LINC01117	11	5.9	1280.7	465.0	2016.1	2143.3
HAGLROS	74.6	59.8	1850.2	924.0	2888.4	3459.5
APTR	38.7	37.0	5526	5246.6	3471.2	4479.2
LINC01611	8.3	5.1	1508.5	1414.3	229.2	675.5
AC108488.4	29.6	26.7	10172	9838	8737.5	9539.8
AC093323.3	319.2	261.1	6356.9	5454.7	5070.7	5425.8
LINC01197	123.6	97.0	8551.2	7910.3	5331.1	5697.2
AC138035.2	6.3	6.0	6428.9	6397.3	5777.8	6687.2
CYP4F26P	95.2	81.1	10928	9835	1916.2	2192.0
AC019186.1	3.3	1.8	142.67	87.4	0	170.7
LINC01521	76.2	65.9	2055.1	1839.7	1580.8	2151.4
LINC00592	5.8	4.1	843.5	154.5	369.59	433.3
LINC01481	10.1	9.2	4126.4	4050.4	3758.8	4282.5

FIRRE	48.7	35.4	738.3	345.7	328.7	405.5
AC005606.15	3.5	3.2	120.1	0	222.7	698.8
AL133245.2	7.6	5.5	247.6	218.3	102.2	184.1
HCG27	8.2	6.4	1033.7	972.6	1017.7	1136.5
LINC00304	1.9	1.8	107.4	32.5	264.2	654.1
AP000569.9	4.2	4.1	864.1	325.5	353.7	483.6
LINC00324	11.6	11.5	103.2	46.2	617.3	848.6
LINC00242	1.9	1.8	143.1	78.1	304.3	404.1
TSIX	15.1	14.7	207.8	170.9	178.4	179.2
OGFRP1	10.6	10.6	1211	1154.3	1294	1285.2
LINC00880	0.4	0.4	4.7	13.9	85.9	76.6
LINC01451	0.7	0.9	177.4	214.7	679.02	535.1
AC195454.1	1.2	1.4	0	74.7	114.5	102.2
AP000654.4	11.8	13.4	2461.5	3156	588.6	575.3
LINC00907	3.4	4.6	322.5	413.8	121.1	104.6
PACERR	2.6	2.8	739.6	1391.5	895.9	736.7
AF064858.8	1.2	1.3	212.1	373.0	231.0	81.0
DSCR9	1.6	1.8	2040.8	2300	2713.7	2488.
MIR34AHG	403.5	462.2	7114.8	7521.8	7881.5	7553.2
C16orf47	3.4	4.5	21.1	151.7	414.2	259.9
LINC01588	4.3	5.5	826.6	985.0	947.1	589.9
LINC01089	209.5	213.4	9056.9	10283	11237	10918.6
SPATA13	268.1	308.7	5879	6766.2	3970.4	3700.5
EWSAT1	8.9	10.5	490.7	548.4	703.6	165.8
MEG9	24.6	33.3	1318.2	1579.5	5263.1	4948.6
LINC01497	1.2	1.8	0	163.9	164.5	0
LINC01134	25.3	42.1	469.6	797.1	540.4	446.5
LINC00426	18.2	27.2	1871.8	2616.7	511.6	358.9
MIAT	20.3	23.8	5146.6	6314.9	5169.7	4701.0
MIATNB	57.0	67.9	5146.6	6314.9	5169.7	4701.0
MIR181A1HG	17.0	33.3	524.8	759.5	500.1	306.2
AC021218.2	4.2	8.3	223.1	607.3	382.4	291.4
LINC00211	6.7	12.8	544.1	1138.6	1058.7	874.4
FTX	84.5	96.7	6934.3	7778.9	6653.9	5764.0
BISPR	85.7	202.1	11207	11468	10313	10123.8
LINC01119	7.1	11.11	663.6	1842.9	685.4	434.1
FALEC	1.2	3.2	420.4	492.3	498.0	495.4
C1orf143	0.7	2.2	779.9	1405.2	282.2	226.1
AC097381.1	2.4	7.4	1663.7	1796.3	690.8	273.1

NEAT1	9133	9552	17167	17770	19738	17763.5
MIR193BHG	6.9	10.5	908.7	1103.1	1596.5	120.9
AC144450.2	15.3	51.2	704.0	1337.8	2240.6	2064.5
AC141930.2	1.5	5.5	704.0	1337.8	2240.6	2064.5
LINC00519	1.0	2.7	183.5	195.7	3652.8	2433.2
CASC15	256.8	319	4835	5889.4	5123.8	3360.7
LINC01268	14.2	21.2	366.7	1148.8	4803.2	2413.9
AC073283.4	2.0	4.5	790.4	770.16	4260.2	1950.9
MEG3	4355	6159	11206	11759	18957	16033.5
AC007879.2	0.7	6.4	369.4	521.6	674.2	114.3
MEG8	19.6	29.0	11206	11759	18957	16033.5
AF127936.9	8.8	16.1	8434.4	12135	9315.6	7400
LINC00640	0	1.8	123.2	1000.9	1540.6	632.8
AC123023.1	6.7	43.9	235.8	2307.2	1329	270.7
AF127936.7	9.5	39.2	8434.4	12135	9315.6	7400
EGOT	1.7	41.5	2.134	2794.3	378.5	105.2
MIR3142HG	50.0	187	177.7	6375.9	5925.1	4301.8

Supplemental Table 2. Information of human donors

Donor ID	Race	Age (Years)	Sex	HbA1c (%)	BMI (kg/m^2)	Health State
1	Hispanic	44	Male	5.2	31.1	Non-DM
2	Asian	64	Male	5.6	30	Non-DM
3	Asian	35	Male	5.6	21.9	Non-DM
4	Caucasian	37	Male	5.5	29.5	Non-DM
5	Hispanic	25	Male	5.4	25.4	Non-DM
6	Caucasian	61	Male	5.6	27	Non-DM
7	Hispanic	35	Male	5.4	24.3	Non-DM
8	Black	23	Male	5.7	20.6	Pre-T2DM
9	Hispanic	48	Female	5.7	34.4	Pre-T2DM
10	Caucasian	32	Male	6	29	Pre-T2DM
11	Caucasian	56	Male	5.2	47.7	Morbid Obesity
12	Asian	28	Male	5.2	38.1	Severe Obesity
13	Hispanic	33	Male	9	28.7	T2DM
14	Caucasian	59	Male	6.8	36.6	T2DM
15	Hispanic	51	Female	9.6	43.3	T2DM

Supplemental Table 3. Mice generated from F1 breeding

No. of litters	Total No. of pups	Genotype	No. of pups	Percentage (%)
25	245	WT	79	32%
		Heterozygous	118	48%
		Homozygous	48	20%

Supplemental Table 4. Mice generated from *leene*-KO and WT littermates

	**WT**	**KO**
**No. of litters**	11	12
**Total No. of pups**	76	92
**Average pups per litter**	7	8
**No. of male**	40	50
**No. of female**	36	42

Supplemental Table 5. Top 10 Candidate LEENE-binding proteins

	RNAct	RPiSeq		
**Gene**	**Prediction Score**	**RF Class**	**SVM**	**FDR**
AEBP2	30.61	0.8	0.77	0.016
RNF216	20.18	0.75	0.9	0.016
CACNB1	18.82	0.9	0.82	0.025
**MYC**	18.63	0.85	0.89	0.013
FARP1	16.03	0.85	0.9	0.008
ZBTB47	15.77	0.95	0.84	0.002
PAK3	15.64	0.85	0.89	0.002
TSPYL2	15.63	0.8	0.74	0.01
BMP2K	15.06	0.9	0.89	0.003
ATMIN	15.04	0.8	0.8	0.002

Supplemental Table 6.
Top 20 TFs involved in LEENE-regulome identified from IPA upstream analysis

Rank	TF	Target Molecules in Dataset	# of Target Genes
1	TP53	ABCG2,ADA,ADORA2B,AK4,ALDH1A2,ANTXR1,BOK,CKB,COL4A1,COL4A2,CSK,CTSD,CXCL12,CYP1A1,DOK1,DPYSL4,E2F1,EDN1,ETFA,F2R,FABP5,FAM83D,FKBP1B,GAS6,GATA6,GJA1,GLI1,GSN,IER3,IGFBP2,IGFBP4,IL17RA,IL27RA,JUNB,KDR,KIF22,KIF23,LAMP1,LPP,LTBP1,MCM6,MYL9,MYO10,MYOF,NPTX1,NYNRIN,P4HA1,PDE4B,PDGFB,PFKP,PTGER1,PTGS2,RASSF2,RHOB,RPE,SERPINB9,SHISA5,SLC19A1,TNFRSF10D,TNFRSF11A,UBE2T,USO1,YBX1,YPEL3	64
2	TP73	ADA,ADAM17,ADAM23,ADORA2B,ANGPTL4,CTSD,E2F1,EDN1,FKBP1B,GPR137B,IER3,IGFBP4,JAG1,JAG2,KIF22,KIF23,LTBP1,MCM6,PDGFB,PIEZO2,SHISA5,YBX1	22
3	HMGA1	BOK,COL4A1,COL4A2,CSK,DPYSL4,GSN,IER3,IGFBP4,JUNB,NECTIN1,PTGS2,RHOB,SOX4	13
4	NPM1	COL4A1,COL4A2,E2F1,FZD8,GJA1,JAG1,JUNB,MEF2C,NUAK1,PDGFB,PGF,SLC6A8	12
5	HIF1A	ADAM17,ADORA2B,AK4,ANGPTL4,CTSD,CXCL12,CYP1A1,EDN1,FHL3,GATA6,GJA1,H2AC6,IGFBP2,IL17RA,KIFC2,MCM6,MEF2C,P4HA1,PDGFB,PFKP,PGF,PTGS2,SLC35A2,TMEM19	24
6	NKX2-3	AIF1L,ANGPTL4,BMP4,DHX58,EDN1,F2RL1,F2RL3,FBXO6,GMPR,HEY2,PIM3,PLCB1,PTGS2,TNFRSF10D	14
7	CTNNB1	ABCD4,ADAM17,ALDH1A2,BMP4,CENPM,COL4A1,COL4A2,CTHRC1,CXCL12,CYP1A1,DOK1,EDN1,ENC1,F2R,GALC,GJA1,GLI1,GPR137B,HMG20B,IGFBP2,JAG1,KDR,KIF23,LFNG,MAP3K11,MFGE8,NPTX1,PARD3,PDE4B,PTGS2,RPL10A,SOX4,USO1,YPEL3	34
8	SMAD4	ABCG2,ANGPTL4,BMP4,CTSD,DOCK4,EDN1,GJA1,GLI1,IER3,JAG1,JAG2,PDGFB,PTGS2,SHISA5,SMAD3	15
9	COPS5	ADA,BLCAP,CTSD,E2F1,F2R,KIF22,KIF23,MARCKS,MCM6,NME4,PDE4B,PLK3,TK1	13
10	KLF2	BMP4,CD40,COL4A1,EDN1,F2RL1,JUNB,KDR,PGF,PTGS2,RGS3,SERPINB9	11
11	YAP1	ALDH1A2,CXCL12,E2F1,EDN1,IGFBP4,JAK1,KIF23,MCM6,MYL9,P4HA1,PDGFB,PPP1R3B,PTGS2,RANBP1,RPL10A,TK1	16
12	AIP	AHRR,CYP1A1,F2RL1,GNG11,PDE3A,PDE4B	6
13	TCF7L2	ALDH1A2,BMP4,EDN1,EFNB1,ENC1,ENTPD5,GLI1,GSN,IGFBP2,JAG1,LAMP1,PELI1,PIM3,PLEKHG3,PTGS2,TMEM123,TSPAN15	17
14	NANOG	BMP4,E2F1,GATA6,GDF6,GLI1,KDR,LTBP1,NPTX1,SMAD3,SMARCC1	10
15	KLF6	ANGPTL4,ATOX1,CYP1A1,DOCK4,EXT1,GLI1,IL17RA,JUNB,PDE4B,PFKP,PTGS2,RHOB,SMAD3	13
16	TP63	ADA,BMP4,BOK,COL4A1,CTSD,DOK1,E2F1,F2R,IER3,IGFBP2,JAG1,JAG2,JUNB,KIF23,MFGE8,MRPL41,NCS1,SMAD3,SOX4	19
17	MEOX1	BMP4,GLI1,MEF2C	3
18	SOX7	COL4A1,COL4A2,FLT4,KDR,MFGE8,SOX4	6
19	TCF20	C2CD4C,EPHB6,LFNG,PATJ,RGS20,TSPAN9	6
20	BHLHE40	ADORA2B,AK4,CD9,CSK,EXT1,IER3,IL17RA,MEF2C,P4HA1,PDE4B,PFKP,PTGS2,RPE,SLC7A2	14

Supplemental Table 7. Sequences of primers

Gene/Primer ID	Species	Assay	Sequence
P1	mouse	Genotyping	ATTTTCAAGCAATGAGCTAGGG
P2	mouse	Genotyping	TCAATCTCATTCAAACGACCAC
P3	mouse	Genotyping	TCACCAGTCCAATCTGAGCC
36B4	mouse	RT-PCR	Forward AGATTCGGGATATGCTGTTGGC<br>Reverse TCGGGTCCTAGACCAGTGTTC
Kdr	mouse	RT-PCR	Forward TCCAGAATCCTCTTCCATGC<br>Reverse AAACCTCCTGCAAGCAAATG
leene	mouse	RT-PCR	Forward GGACCTCTGGCTAAGGTGAG<br>Reverse TCCTTGCTTGTCCTGTCAGT
Peli2	mouse	RT-PCR	Forward AGGACATCACAGCATATCGTACA<br>Reverse CGAAGTCAATGGGGCTTTCTG
Ktn1	mouse	RT-PCR	Forward TGCATCAAAGATCCCAGGCAA<br>Reverse TGCTCTTCGCTTCCATTTTTAGA
Gm49302	mouse	RT-PCR	Forward CTGGCACCCACTAGGATGAC<br>Reverse AGCAAATGGTCCCTTGGGTT
Gm35360	mouse	RT-PCR	Forward AGCCGTTGGAAAAGGGTGAA<br>Reverse CTGAGAAGGTGCTACGGGTG
LEENE	human	RT-PCR	Forward TTTCCCTCTTTGGGGTCTCA<br>Reverse GCCCTTTGATGAGTGAGTCG
eNOS	human	RT-PCR	Forward TGATGGCGAAGCGAGTGAAG<br>Reverse ACTCATCCATACACAGGACCC
KDR	human	RT-PCR	Forward GTGATCGGAAATGACACTGGAG<br>Reverse CATGTTGGTCACTAACAGAAGCA
ACTB	human	RT-PCR	Forward CATGTACGTTGCTATCCAGGC<br>Reverse CTCCTTAATGTCACGCACGAT
LEO1	human	RT-PCR	Forward CGGATATGGAGGATCTCTTCGG<br>Reverse CAGAGGCATTACTGCCAGAGG
KDR	human	ChIP-qPCR	Forward ACACATTGACCGCTCTCCC<br>Reverse GCTCTAGAGTTTCGGCACCAG
PGF	human	ChIP-qPCR	Forward TCCGTCGATGCAGTTTCCTC<br>Reverse GCTCAGTCCCTGAAACCCAG
eNOS	human	ChIRP-qPCR	Forward GCCGAACACCAAATCTCCAAC<br>Reverse AGCCCTGCCAAGAATGATGC
LEENE	human	ChIRP-qPCR	Forward TCCCATGACATGCGGAGATT<br>Reverse TCCCTGAGTCTTGGGTTCTTC
KDR	human	ChIRP-qPCR	Forward ACACATTGACCGCTCTCCC<br>Reverse GCTCTAGAGTTTCGGCACCAG
sgRNAs	mouse		GCTGCGATCCGAACAGTGAG<br>TCGATCCTCATACATTTCAT
PGF	human	RT-PCR	Forward CAGAGGTGGAAGTGGTACCCTTCC<br>Reverse CGGATCTTTAGGAGCTGCATGGTGAC

PELI2	human	RT-PCR	Forward AATAAGGAGCCAGTGAAATACGG<br>Reverse CCGCTTGTAGAGGGCAAATC
KTN1	human	RT-PCR	Forward AAGGAAAGGCAGCAACAGGT<br>Reverse CTGACCCTGAAGTTCCAGCC
MYC	human	RT-PCR	Forward GCTGCTTAGACGCTGGATTT<br>Reverse CTCCTCCTCGTCGCAGTAGA

Supplemental References

1. Tang X, Miao Y, Luo Y, Sriram K, Qi Z, Lin FM, et al. Suppression of Endothelial AGO1 Promotes Adipose Tissue Browning and Improves Metabolic Dysfunction. *Circulation.* 2020;142(4):365-79.
2. Nam D, Ni CW, Rezvan A, Suo J, Budzyn K, Llanos A, et al. Partial carotid ligation is a model of acutely induced disturbed flow, leading to rapid endothelial dysfunction and atherosclerosis. *American journal of physiology Heart and circulatory physiology.* 2009;297(4):H1535-43.
3. Miao Y, Ajami NE, Huang TS, Lin FM, Lou CH, Wang YT, et al. Enhancer-associated long non-coding RNA LEENE regulates endothelial nitric oxide synthase and endothelial function. *Nature communications.* 2018;9(1):292.
4. Chen Z, Lai TC, Jan YH, Lin FM, Wang WC, Xiao H, et al. Hypoxia-responsive miRNAs target argonaute 1 to promote angiogenesis. *The Journal of clinical investigation.* 2013;123(3):1057-67.
5. Lai CH, Chen AT, Burns AB, Sriram K, Luo Y, Tang X, et al. RAMP2-AS1 Regulates Endothelial Homeostasis and Aging. *Frontiers in cell and developmental biology.* 2021;9:635307.
6. Eglinger J, Karsjens H, and Lammert E. Quantitative assessment of angiogenesis and pericyte coverage in human cell-derived vascular sprouts. *Inflammation and regeneration.* 2017;37:2.
7. Das S, Senapati P, Chen Z, Reddy MA, Ganguly R, Lanting L, et al. Regulation of angiotensin II actions by enhancers and super-enhancers in vascular smooth muscle cells. *Nature communications.* 2017;8(1):1467.
8. Zhang G, Wang X, Li C, Li Q, An YA, Luo X, et al. Integrated Stress Response Couples Mitochondrial Protein Translation With Oxidative Stress Control. *Circulation.* 2021;144(18):1500-15.
9. Chu C, Quinn J, and Chang HY. Chromatin isolation by RNA purification (ChIRP). *Journal of visualized experiments : JoVE.* 2012(61).
10. He M, Huang TS, Li S, Hong HC, Chen Z, Martin M, et al. Atheroprotective Flow Upregulates ITPR3 (Inositol 1,4,5-Trisphosphate Receptor 3) in Vascular Endothelium via KLF4 (Krüppel-Like Factor 4)-Mediated Histone Modifications. *Arteriosclerosis, thrombosis, and vascular biology.* 2019;39(5):902-14.
